# Protein secondary structure prediction for a single-sequence using hidden semi-Markov models

**DOI:** 10.1186/1471-2105-7-178

**Published:** 2006-03-30

**Authors:** Zafer Aydin, Yucel Altunbasak, Mark Borodovsky

**Affiliations:** 1 School of Electrical and Computer Engineering, Georgia Institute of Technology, Atlanta, GA 30332-0250, USA; 2 School of Biology, the Wallace H. Coulter Department of Biomedical Engineering and the Center for Bioinformatics and Computational Biology, Georgia Institute of Technology, Atlanta, GA 30332-0230, USA

## Abstract

**Background:**

The accuracy of protein secondary structure prediction has been improving steadily towards the 88% estimated theoretical limit. There are two types of prediction algorithms: Single-sequence prediction algorithms imply that information about other (homologous) proteins is not available, while algorithms of the second type imply that information about homologous proteins is available, and use it intensively. The single-sequence algorithms could make an important contribution to studies of proteins with no detected homologs, however the accuracy of protein secondary structure prediction from a single-sequence is not as high as when the additional evolutionary information is present.

**Results:**

In this paper, we further refine and extend the hidden semi-Markov model (HSMM) initially considered in the BSPSS algorithm. We introduce an improved residue dependency model by considering the patterns of statistically significant amino acid correlation at structural segment borders. We also derive models that specialize on different sections of the dependency structure and incorporate them into HSMM. In addition, we implement an iterative training method to refine estimates of HSMM parameters. The three-state-per-residue accuracy and other accuracy measures of the new method, IPSSP, are shown to be comparable or better than ones for BSPSS as well as for PSIPRED, tested under the single-sequence condition.

**Conclusions:**

We have shown that new dependency models and training methods bring further improvements to single-sequence protein secondary structure prediction. The results are obtained under cross-validation conditions using a dataset with no pair of sequences having significant sequence similarity. As new sequences are added to the database it is possible to augment the dependency structure and obtain even higher accuracy. Current and future advances should contribute to the improvement of function prediction for orphan proteins inscrutable to current similarity search methods.

## Background

Accurate prediction of the regular elements of protein 3D structure is important for precise prediction of the whole 3D structure. A protein secondary structure prediction algorithm assigns to each amino acid a structural state from a 3-letter alphabet {H, E, L} representing the *α*-helix, *β*-strand and loop, respectively. Prediction of function via sequence similarity search for new proteins (function annotation transfer) should be facilitated by a more accurate prediction of secondary structure since structure is more conserved than sequence.

Algorithms of protein secondary structure prediction frequently employ neural networks [1-7], support vector machines [8-13] and hidden Markov models [14-16]. Parameters of the algorithm have to be defined by machine learning, therefore algorithm development and assessment usually contains four steps. The first one is a statistical analysis to identify the most informative correlations and patterns. The second one is the creation of a model that represents dependencies between structure and sequence elements. In the third step, the model parameters are derived from a training set. Finally, in the fourth step, the algorithm prediction accuracy is assessed on test samples (sets) with known structure.

There are two types of protein secondary structure prediction algorithms. A single-sequence algorithm does not use information about other (homologous) proteins. The algorithm should be suitable for a sequence with no similarity to any other protein sequence. Algorithms of another type are explicitly using sequences of homologous proteins, which often have similar structures. The prediction accuracy of such an algorithm should be higher than one of a single-sequence algorithm due to incorporation of additional evolutionary information from multiple alignments [17].

The estimated theoretical limit of the accuracy of secondary structure assignment from experimentally determined 3D structure is 88% [18]. The accuracy (see formal accuracy definition below) of the best current single-sequence prediction methods is below 70% [19].  BSPSS [14], SIMPA [20], SOPM [21], and GOR V [22] are examples of single-sequence prediction algorithms. Among the current best methods that use evolutionary information (multiple alignments, PSI-BLAST profiles), one can mention PSIPRED [1], Porter [23], SSpro [24], APSSP2 [2], SVMpsi [9], PHDpsi [25], JPRED2 [4] and PROF [26]. For instance, the prediction accuracy of Porter was shown to be as high as 80.4% [27]. The joint utilization of methods that specialize on single-sequence prediction and methods using homology information will definitely improve the prediction performance.

Single-sequence algorithms for protein secondary structure prediction are important because a significant percentage of the proteins identified in genome sequencing projects have no detectable sequence similarity to any known protein [28,29]. Particularly in sequenced prokaryotic genomes, about a third of the protein coding genes are annotated as encoding hypothetical proteins lacking similarity to any protein with a known function [30]. Also, out of the 25,000 genes believed to be present in the human genome, no more than 40–60% can be assigned a functional role based on similarity to known proteins [31,32]. For a larger picture, the Pfam database allows one to get information on the distribution of proteins with known functional domains in three domains of life (Table 1).

From the structure prediction standpoint, it is important that two or more hypothetical proteins may bear similarity with each other, in which case it still would be possible to incorporate evolutionary information in a structure prediction algorithm. However, many hypothetical proteins would not have detectable similarity to any protein at all. Such "orphan" proteins may represent a sizeable portion of a proteome, as it is shown in Table 2 representing three newly sequenced genomes.

For an orphan protein, any method of secondary structure prediction performs as a single-sequence method. Developing better methods of protein secondary structure prediction from single-sequence has a definite merit as it helps improving the functional annotation of orphan proteins. In this work, we describe a new algorithm for protein secondary structure prediction, which develops further the model suggested by Schmidler *et al*. [14]. We consider the protein secondary structure prediction as a problem of maximization of *a posteriori *probability of a structure, given primary sequence, as defined by a hidden semi-Markov model (HSMM). To determine the architecture of this HSMM, we performed a statistical analysis identifying the most informative correlations between sequence and structure variables. We specifically considered correlations at proximal positions of structural segments and dependencies to upstream and downstream residues. Finally, we proceeded with an iterative estimation of the HSMM parameters.

## Results and discussion

We first compared the performances of BSPSS [14] and IPSSP in strict jacknife conditions. In our computations, we used the EVA set of "sequence-unique" proteins derived from the PDB database (see the Methods section). We removed sequences shorter than 30 amino acids and arrived to a set of 2720 proteins. The performances of IPSSP and BSPSS were evaluated by a leave-one-out cross validation experiment (jacknife procedure) on this reduced set.

Then we evaluated and compared the performances of BSPSS, IPSSP and PSIPRED on the set of 81 CASP6 targets (see the Methods section) that are available in the PDB. This evaluation is at the "single-sequence condition" implying no additional evolutionary information is available. We used the software "PSIPRED_single", version 2.0, which uses a set of fixed weight matrices in the neural network and does not employ PSI-BLAST profiles. This program was downloaded from the PSIPRED server [33] with the available training data (see the Methods section). We used the same training set to estimate the parameters of BSPSS and IPSSP.

To reduce eight secondary structure states used in the DSSP notation to three, it is possible to use different conversion rules. Here we considered the following three rules: (i) H, G and I to H; E, B to E; all other states to L, (ii) H, G to H; E, B to E; all other states to L, (iii) H to H; E to E; all other states to L. The first rule is also known as the 'EHL' mapping [34,35], the second rule is the one used in PSIPRED [1] and earlier outlined by Rost and Sander [36], while, finally, the third rule is the common 'CK' mapping, which is the one used in BSPSS and other methods [17,37,38]. We also analyzed the effect of making further adjustments after applying either of the three conversion rules. We used the adjustments proposed by Frishman and Argos [39] that lead to a secondary structure sequence with the minimum *β*-strand length of 3 and the minimum *α*-helix length of 5. In our simulations, we used *D *= 50 for the maximum allowed segment length. This value is sufficiently large to cover almost all observed uniform secondary structure segments (see the Bayesian formulation section). For the IPSSP method, we performed 2 iterations and used a percentage threshold value of 35% in the dataset reduction step (see the Iterative model training section).

### Performance measures

We have compared the performances of the methods in terms of four measures: the Sensitivity, Specificity, Matthew's correlation coefficient and Segment Overlap score. We use the three-state-per-residue accuracy (*Q*_3_), defined in Eq. 1 as the overall sensitivity measure:

Q3(%)=NcN×100.     (1)
 MathType@MTEF@5@5@+=feaafiart1ev1aaatCvAUfKttLearuWrP9MDH5MBPbIqV92AaeXatLxBI9gBaebbnrfifHhDYfgasaacH8akY=wiFfYdH8Gipec8Eeeu0xXdbba9frFj0=OqFfea0dXdd9vqai=hGuQ8kuc9pgc9s8qqaq=dirpe0xb9q8qiLsFr0=vr0=vr0dc8meaabaqaciaacaGaaeqabaqabeGadaaakeaacqWGrbqudaWgaaWcbaGaeG4mamdabeaakmaabmaabaGaeiyjaucacaGLOaGaayzkaaGaeyypa0ZaaSaaaeaacqWGobGtdaWgaaWcbaGaem4yamgabeaaaOqaaiabd6eaobaacqGHxdaTcqaIXaqmcqaIWaamcqaIWaamcqGGUaGlcaWLjaGaaCzcamaabmaabaGaeGymaedacaGLOaGaayzkaaaaaa@3FC5@

**Figure 1:**
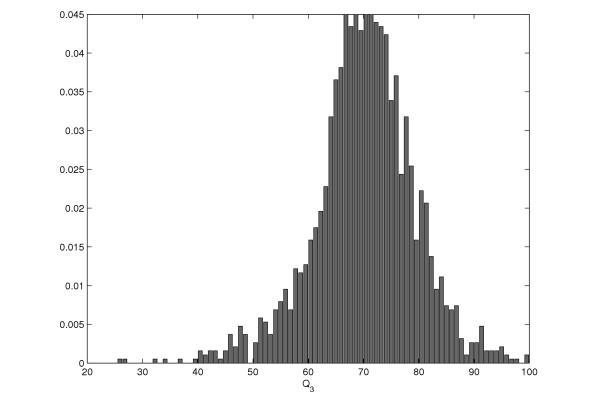
Distribution of the prediction accuracy, *Q*_3_(%), over different amino acid sequences in the dataset.

Here, *N*_*c *_is the total number of residues with correctly predicted secondary structure, *N *is the total number of observed amino acids. The same measure can be used for each type of secondary structure, *Q*_*α*_, *Q*_*β *_and *Q*_*L *_(Eq. 2):

Qi(%)=NciNi×100,     (2)
 MathType@MTEF@5@5@+=feaafiart1ev1aaatCvAUfKttLearuWrP9MDH5MBPbIqV92AaeXatLxBI9gBaebbnrfifHhDYfgasaacH8akY=wiFfYdH8Gipec8Eeeu0xXdbba9frFj0=OqFfea0dXdd9vqai=hGuQ8kuc9pgc9s8qqaq=dirpe0xb9q8qiLsFr0=vr0=vr0dc8meaabaqaciaacaGaaeqabaqabeGadaaakeaacqWGrbqudaWgaaWcbaGaemyAaKgabeaakmaabmaabaGaeiyjaucacaGLOaGaayzkaaGaeyypa0ZaaSaaaeaacqWGobGtdaqhaaWcbaGaem4yamgabaGaemyAaKgaaaGcbaGaemOta40aaWbaaSqabeaacqWGPbqAaaaaaOGaey41aqRaeGymaeJaeGimaaJaeGimaaJaeiilaWIaaCzcaiaaxMaadaqadaqaaiabikdaYaGaayjkaiaawMcaaaaa@4318@

where Nci
 MathType@MTEF@5@5@+=feaafiart1ev1aaatCvAUfKttLearuWrP9MDH5MBPbIqV92AaeXatLxBI9gBaebbnrfifHhDYfgasaacH8akY=wiFfYdH8Gipec8Eeeu0xXdbba9frFj0=OqFfea0dXdd9vqai=hGuQ8kuc9pgc9s8qqaq=dirpe0xb9q8qiLsFr0=vr0=vr0dc8meaabaqaciaacaGaaeqabaqabeGadaaakeaacqWGobGtdaqhaaWcbaGaem4yamgabaGaemyAaKgaaaaa@30A8@ is the total number of residues with correctly predicted secondary structure of type *i*, and *N*^*i *^is the total number of amino acids observed in conformation of type *i*. The distribution of IPSSP predictions evaluated on the EVA set with respect to the sensitivity measure is shown in Fig. 1.

We first compared the performances of BSPSS and IPSSP on the EVA set. From the results shown in Table 8, there is a 1.9% increase in the overall 3-state prediction accuracy in comparison with BSPSS, when the third conversion rule was used with the length adjustments.

The prediction accuracy of the structural conformation of the residues situated close to structural segment borders (residues located in proximal positions) is measured by sensitivity values computed as overall *Q*_3_*sb *_as well as structure type specific *Q*_*α*_*sb*_, *Q*_*β*_*sb*_, *Q*_*L*_*sb*_. We observed that, the accuracy of IPSSP is better than BSPSS in proximal positions by 1.6% (Table 9).

The specificity measure *SP*_*i *_is defined for individual types of secondary structure as follows:

SPi(%)=NciNpi,     (3)
 MathType@MTEF@5@5@+=feaafiart1ev1aaatCvAUfKttLearuWrP9MDH5MBPbIqV92AaeXatLxBI9gBaebbnrfifHhDYfgasaacH8akY=wiFfYdH8Gipec8Eeeu0xXdbba9frFj0=OqFfea0dXdd9vqai=hGuQ8kuc9pgc9s8qqaq=dirpe0xb9q8qiLsFr0=vr0=vr0dc8meaabaqaciaacaGaaeqabaqabeGadaaakeaacqWGtbWucqWGqbaudaWgaaWcbaGaemyAaKgabeaakmaabmaabaGaeiyjaucacaGLOaGaayzkaaGaeyypa0ZaaSaaaeaacqWGobGtdaqhaaWcbaGaem4yamgabaGaemyAaKgaaaGcbaGaemOta40aa0baaSqaaiabdchaWbqaaiabdMgaPbaaaaGccqGGSaalcaWLjaGaaCzcamaabmaabaGaeG4mamdacaGLOaGaayzkaaaaaa@40CD@

where Npi
 MathType@MTEF@5@5@+=feaafiart1ev1aaatCvAUfKttLearuWrP9MDH5MBPbIqV92AaeXatLxBI9gBaebbnrfifHhDYfgasaacH8akY=wiFfYdH8Gipec8Eeeu0xXdbba9frFj0=OqFfea0dXdd9vqai=hGuQ8kuc9pgc9s8qqaq=dirpe0xb9q8qiLsFr0=vr0=vr0dc8meaabaqaciaacaGaaeqabaqabeGadaaakeaacqWGobGtdaqhaaWcbaGaemiCaahabaGaemyAaKgaaaaa@30C2@ is the total number of amino acids predicted to be in conformation of type *i*. Note that, we do not consider the overall specificity measure *SP*_3_, since its numeric value is the same as *Q*_3_. It was observed in Table 10 that values of *SP*_*α *_and *SP*_*L *_are higher for IPSSP, while *SP*_*β *_value is higher for BSPSS.

The Matthew's correlation coefficient [40] is a single parameter characterizing the extent of a match between the observed and predicted secondary structure. Matthew's correlation is defined for each type of secondary structure as follows:

MCC=TP*TN−FP*FN[(TN+FN)(TN+FP)(TP+FN)(TP+FP)]1/2     (4)
 MathType@MTEF@5@5@+=feaafiart1ev1aaatCvAUfKttLearuWrP9MDH5MBPbIqV92AaeXatLxBI9gBaebbnrfifHhDYfgasaacH8akY=wiFfYdH8Gipec8Eeeu0xXdbba9frFj0=OqFfea0dXdd9vqai=hGuQ8kuc9pgc9s8qqaq=dirpe0xb9q8qiLsFr0=vr0=vr0dc8meaabaqaciaacaGaaeqabaqabeGadaaakeaacqWGnbqtcqWGdbWqcqWGdbWqcqGH9aqpdaWcaaqaaiabdsfaujabdcfaqjabcQcaQiabdsfaujabd6eaojabgkHiTiabdAeagjabdcfaqjabcQcaQiabdAeagjabd6eaobqaamaadmaabaWaaeWaaeaacqWGubavcqWGobGtcqGHRaWkcqWGgbGrcqWGobGtaiaawIcacaGLPaaadaqadaqaaiabdsfaujabd6eaojabgUcaRiabdAeagjabdcfaqbGaayjkaiaawMcaamaabmaabaGaemivaqLaemiuaaLaey4kaSIaemOrayKaemOta4eacaGLOaGaayzkaaWaaeWaaeaacqWGubavcqWGqbaucqGHRaWkcqWGgbGrcqWGqbauaiaawIcacaGLPaaaaiaawUfacaGLDbaadaahaaWcbeqaaiabigdaXiabc+caViabikdaYaaaaaGccaWLjaGaaCzcamaabmaabaGaeGinaqdacaGLOaGaayzkaaaaaa@6180@

For instance, for the *α*-helix, *TP *(true positives) is the number of *α*-helix residues that are correctly predicted. *TN *(true negatives) is the number of residues observed in *β*-strands and loops that are not predicted as *α*-helix. *FP *(false positives) is the number of residues incorrectly predicted in *α*-helix conformation, and finally *FN *(false negatives) is the number of residues observed in *α*-helices but predicted to be either in *β*-strands or loops. All the MCC values shown in Table 11 are higher for IPSSP.

In terms of the Segment Overlap scores, IPSSP performs uniformly better than BSPSS [see Additional file 1]. We also assessed the reliability of predictions (prediction confidence) produced by the methods BSPSS and IPSSP [see Additional file 2]. The results lead us to the conclusion that IPSSP is better than BSPSS in terms of the reliability measures.

To investigate the effect of length adjustments, we converted short *α*-helices and *β*-strands to loops so that the *α*-helix and *β*-strand segments had at least 5 and 3 residues, respectively [39]. We also compared IPSSP and BSPSS using different conversion rules and length adjustments (Table 12). It is seen that IPSSP performs better than BSPSS for each set of rules.

Next, we compared the performances of the three methods BSPSS, IPSSP and, PSIPRED_v2.0 on 81 CASP6 targets that are available in PDB. From the results shown in Table 15, and Table 16, IPSSP is comparable to PSIPRED and is more accurate than BSPSS.

### Improvements over the BSPSS method

In summary, the differences with the BSPSS algorithm proposed by Schmidler *et al*. [14] are as follows. We introduced three residue dependency models (both probabilistic and heuristic) incorporating the statistically significant amino acid correlation patterns at structural segment borders. In these models, we allowed dependencies to positions outside the segments to relax the condition of segment independence. Another novelty of the models is the dependency to downstream positions, which we believe is necessary due to asymmetric correlation patterns observed uniformly in structural segments. To assess the individual performances of the dependency models, we evaluated IPSSP using ℳ
 MathType@MTEF@5@5@+=feaafiart1ev1aaatCvAUfKttLearuWrP9MDH5MBPbIqV92AaeXatLxBI9gBamrtHrhAL1wy0L2yHvtyaeHbnfgDOvwBHrxAJfwnaebbnrfifHhDYfgasaacH8akY=wiFfYdH8Gipec8Eeeu0xXdbba9frFj0=OqFfea0dXdd9vqai=hGuQ8kuc9pgc9s8qqaq=dirpe0xb9q8qiLsFr0=vr0=vr0dc8meaabaqaciaacaGaaeqabaWaaeGaeaaakeaaimaacqWFZestaaa@3790@_1_, ℳ
 MathType@MTEF@5@5@+=feaafiart1ev1aaatCvAUfKttLearuWrP9MDH5MBPbIqV92AaeXatLxBI9gBamrtHrhAL1wy0L2yHvtyaeHbnfgDOvwBHrxAJfwnaebbnrfifHhDYfgasaacH8akY=wiFfYdH8Gipec8Eeeu0xXdbba9frFj0=OqFfea0dXdd9vqai=hGuQ8kuc9pgc9s8qqaq=dirpe0xb9q8qiLsFr0=vr0=vr0dc8meaabaqaciaacaGaaeqabaWaaeGaeaaakeaaimaacqWFZestaaa@3790@_2_, ℳ
 MathType@MTEF@5@5@+=feaafiart1ev1aaatCvAUfKttLearuWrP9MDH5MBPbIqV92AaeXatLxBI9gBamrtHrhAL1wy0L2yHvtyaeHbnfgDOvwBHrxAJfwnaebbnrfifHhDYfgasaacH8akY=wiFfYdH8Gipec8Eeeu0xXdbba9frFj0=OqFfea0dXdd9vqai=hGuQ8kuc9pgc9s8qqaq=dirpe0xb9q8qiLsFr0=vr0=vr0dc8meaabaqaciaacaGaaeqabaWaaeGaeaaakeaaimaacqWFZestaaa@3790@_3_, and ℳ
 MathType@MTEF@5@5@+=feaafiart1ev1aaatCvAUfKttLearuWrP9MDH5MBPbIqV92AaeXatLxBI9gBamrtHrhAL1wy0L2yHvtyaeHbnfgDOvwBHrxAJfwnaebbnrfifHhDYfgasaacH8akY=wiFfYdH8Gipec8Eeeu0xXdbba9frFj0=OqFfea0dXdd9vqai=hGuQ8kuc9pgc9s8qqaq=dirpe0xb9q8qiLsFr0=vr0=vr0dc8meaabaqaciaacaGaaeqabaWaaeGaeaaakeaaimaacqWFZestaaa@3790@_*C*_, where ℳ
 MathType@MTEF@5@5@+=feaafiart1ev1aaatCvAUfKttLearuWrP9MDH5MBPbIqV92AaeXatLxBI9gBamrtHrhAL1wy0L2yHvtyaeHbnfgDOvwBHrxAJfwnaebbnrfifHhDYfgasaacH8akY=wiFfYdH8Gipec8Eeeu0xXdbba9frFj0=OqFfea0dXdd9vqai=hGuQ8kuc9pgc9s8qqaq=dirpe0xb9q8qiLsFr0=vr0=vr0dc8meaabaqaciaacaGaaeqabaWaaeGaeaaakeaaimaacqWFZestaaa@3790@_*C *_is the combination of the three models obtained using an averaging filter. The results in Table 13 show that the combined models improve the overall accuracy by more than 1% when the second conversion rule (H, G to H, E, B to E and all other states to L) is used without length adjustments. Note that, all three models use a five letter alphabet for positions with significantly high correlation measures. The performance obtained when all the hydrophobicity groupings are defined using the three letter alphabet is 0.4% lower (data not shown).

Apart from the more elaborate dependency structure, we introduced an iterative training strategy to refine estimates of model parameters. The individual contributions of the dependency model and the iterative training is given in Table 14. In this table, the method PSSP refers to the IPSSP method without iterative training. To reduce 8 states to 3, the second conversion rule is used without length adjustments. Under this setting, the dependency model improves the overall sensitivity measure by 1.6% as compared to the BSPSS method. The inclusion of the iterative training further improves the results by 0.5%.

### Single-sequence vs. sequence-unique condition

We would like to emphasize that throughout the paper, we use the term single-sequence prediction in its strict meaning, *i.e*. the prediction method does not exploit information about any protein sequence similar to the sequence in question as for a true single-sequence such information does not exist. The "single-sequence" concept should be distinguished from the concept of the "sequence-unique" category. The "sequence-unique" condition requires the absence of significant similarity between proteins in the test and in the training set. However, this condition leaves an opportunity to use the sequence profile information that typically improves the prediction accuracy by several percentage points in comparison with the single-sequence condition, in which such profiles are not available. Indeed, methods such as APSSP2 [2] and SVMpsi [9] achieved values around 78% in the "sequence-unique" category of CASP [41] and CAFASP [42] experiments. Similarly, the SSPAL method [43] was cited [14] to have 71% accuracy in terms of *Q*_3_(%) again in the "sequence-unique" category. Single-sequence condition, as defined, is more stringent. This condition is common for "orphan" proteins, which have no detectable homologs. Improvement of structural prediction under the single-sequence condition should contribute to the improvement of function prediction for orphan proteins, which are not easy targets for functional characterization.

## Conclusions

We have shown that new dependency models and training methods bring further improvements to single-sequence protein secondary structure prediction. The results are obtained under cross-validation conditions using a dataset with no pair of sequences having significant sequence similarity. As new sequences are added to the database it is possible to augment the dependency structure and obtain even higher accuracy.

Typically protein secondary structure prediction methods suffer from low accuracy in predicting *β*-strands, in which non-local correlations have a significant role. In this work, we did not specifically address this problem, but showed that improvements are possible when higher order dependency models are used and significant correlations outside the segments are considered. To achieve substantial improvements in the prediction accuracy, it is necessary to develop models that incorporate long-range interactions in *β*-sheets. The advances in secondary structure prediction should contribute to the improvement of function prediction for orphan proteins inscrutable to current similarity search methods.

## Methods

The representative set of 2482 amino acid sequences (PDB_SELECT) for preliminary statistical analysis were obtained from [44]. The procedure used to generate the PDB_SELECT list was described earlier [45]. In this set, the percentage of identity between any pair of sequences is less than 25%. The 3324 "sequence-unique" proteins were downloaded from the EVA server ftp site, as of 2004_05_09, and the copy of the data were placed at [46]. The proteins in this set, which was used in leave-one-out cross validation experiments were selected to satisfy the condition that percentage of identity between any pair of sequences should not exceed the length dependent threshold *S *(for instance, for sequences longer than 450 amino acids, *S *= 19.5) [47]. CASP6 targets were downloaded from [48], and the PDB definitions were used for the amino acid sequences and secondary structure assignments. PSIPRED training data was downloaded from [49].

### Bayesian formulation

**Figure 2:**
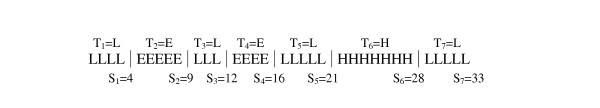
The secondary structure sequence and its representation by structural segments.

The linear sequence that defines a secondary structure of a protein can be described by a pair of vectors (**S**, **T**), where **S **denotes the structural segment end (border) positions and, **T **determines the structural state of each segment (*α*-helix, *β*-strand or loop). For instance, for the secondary structure shown in Fig. 2, **S **= (4, 9, 12, 16, 21, 28, 33) and **T **= (L, E, L, E, L, H, L).

Given a statistical model specifying probabilistic dependencies between sequence and structure elements, the problem of protein secondary structure prediction could be stated as the problem of maximizing the *a posteriori *probability of a structure given the primary sequence. Thus, given the sequence of amino acids, **R**, one has to find the vector (**S**, **T**) with maximum *a posteriori *probability *P*(**S**, **T **| **R**) as defined by an appropriate statistical model. Using Bayes' rule, this probability can be expressed as:

P(S,T|R)=P(R|S,T)P(S,T)P(R)     (5)
 MathType@MTEF@5@5@+=feaafiart1ev1aaatCvAUfKttLearuWrP9MDH5MBPbIqV92AaeXatLxBI9gBaebbnrfifHhDYfgasaacH8akY=wiFfYdH8Gipec8Eeeu0xXdbba9frFj0=OqFfea0dXdd9vqai=hGuQ8kuc9pgc9s8qqaq=dirpe0xb9q8qiLsFr0=vr0=vr0dc8meaabaqaciaacaGaaeqabaqabeGadaaakeaacqWGqbaudaqadaqaaGqabiab=nfatjab=XcaSiab=rfaujab=Xha8jab=jfasbGaayjkaiaawMcaaiabg2da9maalaaabaGaemiuaa1aaeWaaeaacqWFsbGucqWF8baFcqWFtbWucqWFSaalcqWFubavaiaawIcacaGLPaaacqWGqbaudaqadaqaaiab=nfatjab=XcaSiab=rfaubGaayjkaiaawMcaaaqaaiabdcfaqnaabmaabaGae8NuaifacaGLOaGaayzkaaaaaiaaxMaacaWLjaWaaeWaaeaacqaI1aqnaiaawIcacaGLPaaaaaa@4C6D@

where *P*(**R **| **S**, **T**) denotes the likelihood and *P*(**S**, **T**) is the *a priori *probability. Since *P*(**R**) is a constant with respect to (**S**, **T**), maximizing *P*(**S**, **T **| **R**) is equivalent to maximizing *P*(**R **| **S**, **T**)*P*(**S**, **T**). To proceed further, we need models for each of these probabilistic terms. We model the distribution of *a priori *probability *P*(**S**, **T**) as follows:

P(S,T)=∏j=1mP(Tj|Tj−1)P(Sj|Sj−1,Tj).     (6)
 MathType@MTEF@5@5@+=feaafiart1ev1aaatCvAUfKttLearuWrP9MDH5MBPbIqV92AaeXatLxBI9gBaebbnrfifHhDYfgasaacH8akY=wiFfYdH8Gipec8Eeeu0xXdbba9frFj0=OqFfea0dXdd9vqai=hGuQ8kuc9pgc9s8qqaq=dirpe0xb9q8qiLsFr0=vr0=vr0dc8meaabaqaciaacaGaaeqabaqabeGadaaakeaacqWGqbaudaqadaqaaGqabiab=nfatjab=XcaSiab=rfaubGaayjkaiaawMcaaiabg2da9maarahabaGaemiuaa1aaeWaaeaacqWGubavdaWgaaWcbaGaemOAaOgabeaakiabcYha8jabdsfaunaaBaaaleaacqWGQbGAcqGHsislcqaIXaqmaeqaaaGccaGLOaGaayzkaaGaemiuaa1aaeWaaeaacqWGtbWudaWgaaWcbaGaemOAaOgabeaakiabcYha8jabdofatnaaBaaaleaacqWGQbGAcqGHsislcqaIXaqmaeqaaOGaeiilaWIaemivaqLaemOAaOgacaGLOaGaayzkaaGaeiOla4caleaacqWGQbGAcqGH9aqpcqaIXaqmaeaacqWGTbqBa0Gaey4dIunakiaaxMaacaWLjaWaaeWaaeaacqaI2aGnaiaawIcacaGLPaaaaaa@59D7@

Here, *m *denotes the total number of uniform secondary structure segments. *P*(*T*_*j *_| *T*_*j*-1_) is the probability of transition from segment with secondary structure type *T*_*j*-1 _to a segment with secondary structure type *T*_*j*_. Table 3 shows the transition probabilities *P*(*T*_*j *_| *T*_*j*-1_), estimated from a representative set of 2482 "unrelated" proteins (see the Methods section). The third term, *P*(*S*_*j *_| *S*_*j*-1_, *T*_*j*_), reflects the length distribution of the uniform secondary structure segments. It is assumed that

*P*(*S*_*j *_| *S*_*j*-1_, *T*_*j*_) = *P*(*S*_*j *_- *S*_*j*-1 _| *T*_*j*_),     (7)

where *S*_*j *_- *S*_*j*-1 _is equal to the segment length (Fig. 2). The segment length distributions for different types of secondary structure have been determined earlier [50].

The likelihood term *P*(**R **| **S**, **T**) can be written as:

P(R|S,T)=∏j=1mP(R[Sj−1+1:Sj]|S,T)     (8)=∏j=1mP(R[Sj−1+1:Sj]|Sj−1,Sj,Tj)
 MathType@MTEF@5@5@+=feaafiart1ev1aaatCvAUfKttLearuWrP9MDH5MBPbIqV92AaeXatLxBI9gBaebbnrfifHhDYfgasaacH8akY=wiFfYdH8Gipec8Eeeu0xXdbba9frFj0=OqFfea0dXdd9vqai=hGuQ8kuc9pgc9s8qqaq=dirpe0xb9q8qiLsFr0=vr0=vr0dc8meaabaqaciaacaGaaeqabaqabeGadaaakqaaeeqaaiabdcfaqnaabmaabaacbeGae8NuaiLae8hFaWNae83uamLae8hlaWIae8hvaqfacaGLOaGaayzkaaGaeyypa0ZaaebCaeaacqWGqbaudaqadaqaaiab=jfasnaaBaaaleaadaWadaqaaiabdofatnaaBaaameaacqWGQbGAcqGHsislcqaIXaqmaeqaaSGaey4kaSIaeGymaeJaeiOoaOJaem4uam1aaSbaaWqaaiabdQgaQbqabaaaliaawUfacaGLDbaaaeqaaOGaeiiFaWNae83uamLae8hlaWIae8hvaqfacaGLOaGaayzkaaGaaCzcaiaaxMaadaqadaqaaiabiIda4aGaayjkaiaawMcaaaWcbaGaemOAaOMaeyypa0JaeGymaedabaGaemyBa0ganiabg+GivdaakeaacqGH9aqpdaqeWbqaaiabdcfaqnaabmaabaGae8Nuai1aaSbaaSqaamaadmaabaGaem4uam1aaSbaaWqaaiabdQgaQjabgkHiTiabigdaXaqabaWccqGHRaWkcqaIXaqmcqGG6aGocqWGtbWudaWgaaadbaGaemOAaOgabeaaaSGaay5waiaaw2faaaqabaGccqGG8baFcqWGtbWudaWgaaWcbaGaemOAaOMaeyOeI0IaeGymaedabeaakiabcYcaSiabdofatnaaBaaaleaacqWGQbGAaeqaaOGaeiilaWIaemivaq1aaSbaaSqaaiabdQgaQbqabaaakiaawIcacaGLPaaaaSqaaiabdQgaQjabg2da9iabigdaXaqaaiabd2gaTbqdcqGHpis1aaaaaa@7B82@

Here, **R**_[*p*:*q*] _denotes the sequence of amino acid residues with position indices from *p *to *q*. The probability of observing a particular amino acid sequence in a segment adopting a particular type of secondary structure is *P*(R[Sj−1+1:Sj]
 MathType@MTEF@5@5@+=feaafiart1ev1aaatCvAUfKttLearuWrP9MDH5MBPbIqV92AaeXatLxBI9gBaebbnrfifHhDYfgasaacH8akY=wiFfYdH8Gipec8Eeeu0xXdbba9frFj0=OqFfea0dXdd9vqai=hGuQ8kuc9pgc9s8qqaq=dirpe0xb9q8qiLsFr0=vr0=vr0dc8meaabaqaciaacaGaaeqabaqabeGadaaakeaaieqacqWFsbGudaWgaaWcbaWaamWaaeaacqWGtbWudaWgaaadbaGaemOAaOMaeyOeI0IaeGymaedabeaaliabgUcaRiabigdaXiabcQda6iabdofatnaaBaaameaacqWGQbGAaeqaaaWccaGLBbGaayzxaaaabeaaaaa@3A30@ | **S**, **T**). This term is assumed to be equal to *P*(R[Sj−1+1:Sj]
 MathType@MTEF@5@5@+=feaafiart1ev1aaatCvAUfKttLearuWrP9MDH5MBPbIqV92AaeXatLxBI9gBaebbnrfifHhDYfgasaacH8akY=wiFfYdH8Gipec8Eeeu0xXdbba9frFj0=OqFfea0dXdd9vqai=hGuQ8kuc9pgc9s8qqaq=dirpe0xb9q8qiLsFr0=vr0=vr0dc8meaabaqaciaacaGaaeqabaqabeGadaaakeaaieqacqWFsbGudaWgaaWcbaWaamWaaeaacqWGtbWudaWgaaadbaGaemOAaOMaeyOeI0IaeGymaedabeaaliabgUcaRiabigdaXiabcQda6iabdofatnaaBaaameaacqWGQbGAaeqaaaWccaGLBbGaayzxaaaabeaaaaa@3A30@ | *S*_*j*-1_, *S*_*j*_, *T*_*j*_), thus this probability depends only on the secondary structure type of a given segment, and not of adjacent segments. Note that, we ignore the non-local interactions observed in *β*-sheets. This simplification allows us to implement an efficient hidden semi-Markov model.

To elaborate on the segment likelihood terms in Eq. 8, we have to consider the correlation patterns within the segment with uniform secondary structure. These patterns reflect the secondary structure specific physico-chemical interactions. For instance, *α*-helices are strengthened by hydrogen bonding between amino acid pairs situated at specific distances. To correctly define the likelihood term we should also pay attention to proximal positions, typically the four initial and the four final positions of a secondary structure segment. In particular, *α*-helices include capping boxes, where the hydrogen bonding patterns and side-chain interactions are different from the internal positions [51,52]. The observed distributions of amino acid frequencies in proximal (capping boxes) and internal positions of *α*-helix segments are depicted in Schmidler *et al*. [14], and show noticeably distinct patterns.

Presence of this inhomogeneity in the statistical model leads to the following expression for *P*(R[Sj−1+1:Sj]
 MathType@MTEF@5@5@+=feaafiart1ev1aaatCvAUfKttLearuWrP9MDH5MBPbIqV92AaeXatLxBI9gBaebbnrfifHhDYfgasaacH8akY=wiFfYdH8Gipec8Eeeu0xXdbba9frFj0=OqFfea0dXdd9vqai=hGuQ8kuc9pgc9s8qqaq=dirpe0xb9q8qiLsFr0=vr0=vr0dc8meaabaqaciaacaGaaeqabaqabeGadaaakeaaieqacqWFsbGudaWgaaWcbaWaamWaaeaacqWGtbWudaWgaaadbaGaemOAaOMaeyOeI0IaeGymaedabeaaliabgUcaRiabigdaXiabcQda6iabdofatnaaBaaameaacqWGQbGAaeqaaaWccaGLBbGaayzxaaaabeaaaaa@3A30@ | *S*_*j*_, *S*_*j*-1_, *T*_*j*_):

P(R[Sj−1+1:Sj]|Sj,Sj−1,Tj)=PN1(Rkb+1)∏i=kb+2lN+kbPNi−kb(Ri|Ri−1,…,Rkb+1)     (9)×∏i=lN+kb+1kn−lCPInt(Ri|Ri−1,…,Rkb+1)×∏i=−lC+10PC1−i(Ri+kn|Ri+kn−1,…,Rkb+1).
 MathType@MTEF@5@5@+=feaafiart1ev1aaatCvAUfKttLearuWrP9MDH5MBPbIqV92AaeXatLxBI9gBaebbnrfifHhDYfgasaacH8akY=wiFfYdH8Gipec8Eeeu0xXdbba9frFj0=OqFfea0dXdd9vqai=hGuQ8kuc9pgc9s8qqaq=dirpe0xb9q8qiLsFr0=vr0=vr0dc8meaabaqaciaacaGaaeqabaqabeGadaaakqaaeeqaaiabdcfaqjabcIcaOGqabiab=jfasnaaBaaaleaadaWadaqaaiabdofatnaaBaaameaacqWGQbGAcqGHsislcqaIXaqmaeqaaSGaey4kaSIaeGymaeJaeiOoaOJaem4uam1aaSbaaWqaaiabdQgaQbqabaaaliaawUfacaGLDbaaaeqaaOWaaqqaaeaacqWGtbWudaWgaaWcbaGaemOAaOgabeaakiabcYcaSiabdofatnaaBaaaleaacqWGQbGAcqGHsislcqaIXaqmaeqaaOGaeiilaWIaemivaq1aaSbaaSqaaiabdQgaQbqabaaakiaawEa7aiabcMcaPiabg2da9iabdcfaqnaaBaaaleaacqWGobGtdaWgaaadbaGaeGymaedabeaaaSqabaGcdaqadaqaaiabdkfasnaaBaaaleaacqWGRbWAdaWgaaadbaGaemOyaigabeaaliabgUcaRiabigdaXaqabaaakiaawIcacaGLPaaadaqeWbqaaiabdcfaqnaaBaaaleaacqWGobGtdaWgaaadbaGaemyAaKgabeaaliabgkHiTiabdUgaRnaaBaaameaacqWGIbGyaeqaaaWcbeaakmaabmaabaGaemOuai1aaSbaaSqaaiabdMgaPbqabaGccqGG8baFcqWGsbGudaWgaaWcbaGaemyAaKMaeyOeI0IaeGymaedabeaakiabcYcaSiablAciljabcYcaSiabdkfasnaaBaaaleaacqWGRbWAdaWgaaadbaGaemOyaigabeaaliabgUcaRiabigdaXaqabaaakiaawIcacaGLPaaacaWLjaGaaCzcamaabmaabaGaeGyoaKdacaGLOaGaayzkaaaaleaacqWGPbqAcqGH9aqpcqWGRbWAdaWgaaadbaGaemOyaigabeaaliabgUcaRiabikdaYaqaaiabdYgaSnaaBaaameaacqWGobGtaeqaaSGaey4kaSIaem4AaS2aaSbaaWqaaiabdkgaIbqabaaaniabg+GivdaakeaacqGHxdaTdaqeWbqaaiabdcfaqnaaBaaaleaacqWGjbqscqWGUbGBcqWG0baDaeqaaOWaaeWaaeaacqWGsbGudaWgaaWcbaGaemyAaKgabeaakiabcYha8jabdkfasnaaBaaaleaacqWGPbqAcqGHsislcqaIXaqmaeqaaOGaeiilaWIaeSOjGSKaeiilaWIaemOuai1aaSbaaSqaaiabdUgaRnaaBaaameaacqWGIbGyaeqaaSGaey4kaSIaeGymaedabeaaaOGaayjkaiaawMcaaaWcbaGaemyAaKMaeyypa0JaemiBaW2aaSbaaWqaaiabd6eaojabgUcaRiabdUgaRnaaBaaabaGaemOyaigabeaacqGHRaWkcqaIXaqmaeqaaaWcbaGaem4AaS2aaSbaaWqaaiabd6gaUbqabaWccqGHsislcqWGSbaBdaWgaaadbaGaem4qameabeaaa0Gaey4dIunaaOqaaiabgEna0oaarahabaGaemiuaa1aaSbaaSqaaiabdoeadnaaBaaameaacqaIXaqmcqGHsislcqWGPbqAaeqaaaWcbeaakmaabmaabaGaemOuai1aaSbaaSqaaiabdMgaPjabgUcaRiabdUgaRnaaBaaameaacqWGUbGBaeqaaaWcbeaakiabcYha8jabdkfasnaaBaaaleaacqWGPbqAcqGHRaWkcqWGRbWAdaWgaaadbaGaemOBa4gabeaaliabgkHiTiabigdaXaqabaGccqGGSaalcqWIMaYscqGGSaalcqWGsbGudaWgaaWcbaGaem4AaS2aaSbaaWqaaiabdkgaIbqabaWccqGHRaWkcqaIXaqmaeqaaaGccaGLOaGaayzkaaGaeiOla4caleaacqWGPbqAcqGH9aqpcqGHsislcqWGSbaBdaWgaaadbaGaem4qameabeaaliabgUcaRiabigdaXaqaaiabicdaWaqdcqGHpis1aaaaaa@E2CA@

Here, the first and third sub-products represent the probability of observing *l*_*N *_and *l*_*C *_specific amino acids at the segment's N-terminal and C-terminal, respectively. The second sub-product defines the observation probability of given amino acids in the segment's internal positions. Note that, *k*_*b *_and *k*_*e *_designate *S*_*j*-1 _+ 1 and *S*_*j*_, respectively. The probabilistic expression (9) is generic for *α*-helices, *β*-strands and loops. Formula (9) assumes that, the probabilistic model is fully dependent within a segment, *i.e*. observation of an amino acid at a given position depends on all previous amino acids within the segment. However, at this time, the Protein Data Bank (PDB, [53]) does not have a sufficient amount of experimental data to reliably estimate all the parameters of a fully dependent model. Therefore, it is important to reduce the dependency structure and keep only the most important correlations. In order to achieve this goal, we performed the statistical analysis described in the following section.

### Correlation patterns of amino acids

Amino acids have distinct propensities for the adoption of secondary structure conformations [54]. These propensities are in the heart of many secondary structure prediction methods [51,52,55-62]. Our goal is to come up with a dependency pattern that is comprehensive enough to capture the essential correlations yet simple enough in terms of the number of model parameters to allow reliable parameter estimation from the available training data. Therefore, we performed a *χ*^2^-test to identify the most significant correlations between amino acid pairs located in adjacent and non-adjacent positions for each type of secondary structure segments. The *χ*^2^-test compared empirical distribution of amino acid pairs with the respective product of marginal distributions. Therefore, a 20 × 20 contingency table was computed for the frequencies of possible amino acid pairs observed in different structural states. In this test, the threshold was computed as 404.6 for a statistical significance level of 0.05.

We first considered the correlations between amino acid pairs at various separation distances (Table 4). In *α*-helix segments, a residue at position *i *is highly correlated with residues at positions *i *- 2, *i *- 3 and *i *- 4. Similarly, a *β*-strand residue had its highest correlations with residues at positions *i *- 1, *i *- 2, and a loop residue had its most significant correlation with a residue at position *i *- 1. The test statistics for the remaining pairs were above the threshold for statistical significance but these values were considerably lower than the ones listed above. The dependencies that were identified by the statistical analysis are in agreement with the well known physical nature of the secondary structure conformations.

Next, we analyzed proximal positions and a representative set of internal positions. Frequency patterns in proximal positions deviate from the patterns observed in internal positions [51,58]. For a better quantification, we computed the Kullback-Liebler (KL) distance between probability distributions of the proximal and internal positions (Table 5). The observation that the KL distance is significantly higher for positions closer to segment borders suggests that amino acids in proximal locations have significantly different distributions from those at internal regions.

Finally, we performed a *χ*^2^-test for proximal positions to identify the correlations between amino acid pairs at various separation distances. As can be seen from the results for *α*-helix segments (Table 6), the general assumption of segment independence does not hold as statistically significant correlations were observed between residues situated on both sides of the segment borders. For instance, the second amino acid *i *in the *α*-helix N-terminal significantly correlates with the previous amino acid at position *i *- 2, which is outside the segment. This correlation can be caused by physical interactions between nearby residues [51]. Also, the strength of correlation for the *i *+ (*downstream*) residues was different from the strength observed for *i *- (*upstream*) residues (Table 6). This fact indicates an asymmetry in correlation behavior for *i*+ and *i*- residues. The parameters of position specific correlations were also computed for *β*-strand and loop segments (data not shown).

A similar asymmetry in the correlation patterns was also observed in internal positions (data not shown). For instance, for *α*-helices, the *i*^*th *^residue in an internal position is highly correlated with the *i *- 2^*th*^, *i *- 3^*th*^, *i *- 4^*th*^, *i *+ 2^*th *^and *i *+ 4^*th *^residues. The parameters of correlation between *i*^*th*^and *i *- 2^*th *^residues is different from the parameters of correlation between *i*^*th *^and *i *+ 2^*th *^residues.

In the next section, we will refine the probabilistic model needed to determine *P*(R[Sj−1+1:Sj]
 MathType@MTEF@5@5@+=feaafiart1ev1aaatCvAUfKttLearuWrP9MDH5MBPbIqV92AaeXatLxBI9gBaebbnrfifHhDYfgasaacH8akY=wiFfYdH8Gipec8Eeeu0xXdbba9frFj0=OqFfea0dXdd9vqai=hGuQ8kuc9pgc9s8qqaq=dirpe0xb9q8qiLsFr0=vr0=vr0dc8meaabaqaciaacaGaaeqabaqabeGadaaakeaaieqacqWFsbGudaWgaaWcbaWaamWaaeaacqWGtbWudaWgaaadbaGaemOAaOMaeyOeI0IaeGymaedabeaaliabgUcaRiabigdaXiabcQda6iabdofatnaaBaaameaacqWGQbGAaeqaaaWccaGLBbGaayzxaaaabeaaaaa@3A30@ | *S*_*j*-1_, *S*_*j*_, *T*_*j*_) using the most significant correlations identified by the statistical analysis.

### Reduced dependency model

Correlation analysis allows one to reduce the alphabet size in the likelihood expression (Eq. 9) by selecting only the most significant correlations. The dependence patterns revealed by the statistical analysis are shown in Table 7 divided into panels for *α*-helix (H), *β*-strand (E), and loop (L) structures. To reduce the dimension of the parameter space, we grouped the amino acids into three and five hydrophobicity classes. We used five classes only for those positions, which have significantly high correlation measures. In Table 7, hi−13
 MathType@MTEF@5@5@+=feaafiart1ev1aaatCvAUfKttLearuWrP9MDH5MBPbIqV92AaeXatLxBI9gBaebbnrfifHhDYfgasaacH8akY=wiFfYdH8Gipec8Eeeu0xXdbba9frFj0=OqFfea0dXdd9vqai=hGuQ8kuc9pgc9s8qqaq=dirpe0xb9q8qiLsFr0=vr0=vr0dc8meaabaqaciaacaGaaeqabaqabeGadaaakeaacqWGObaAdaqhaaWcbaGaemyAaKMaeyOeI0IaeGymaedabaGaeG4mamdaaaaa@325E@ stands for the dependency of an amino acid at position *i *to the hydrophobicity class of an amino acid at position *i *- 1, and the superscript 3 represents the total number of hydrophobicity classes.

To better characterize the features that define the secondary structure, we distinguished positions within a segment as well as segments with different lengths. We identified as proximal positions those in which the amino acid frequency distributions significantly deviate from ones in internal positions in terms of the KL distance (Table 5). Based on the available training data, we chose 6 proximal positions (N1-N4, C1-C2) for *α*-helices, 4 proximal positions (N1-N2, C1-C2) for *β*-strands, and 8 proximal positions (N1-N4, C1-C4) for loops. The remaining positions are defined as internal positions (Int). In addition to position specific dependencies, we derived separate patterns for segments with different lengths. Table 7 shows the dependence patterns for segments longer than *L *residues, where *L *is 5 for *α*-helices, and 3 for *β*-strands and loops. For shorter segments, we selected a representative set of patterns from Table 7 according to the available training data. 

To fully utilize the dependency structure, we found it useful to derive three separate dependency models. The first model, ℳ
 MathType@MTEF@5@5@+=feaafiart1ev1aaatCvAUfKttLearuWrP9MDH5MBPbIqV92AaeXatLxBI9gBamrtHrhAL1wy0L2yHvtyaeHbnfgDOvwBHrxAJfwnaebbnrfifHhDYfgasaacH8akY=wiFfYdH8Gipec8Eeeu0xXdbba9frFj0=OqFfea0dXdd9vqai=hGuQ8kuc9pgc9s8qqaq=dirpe0xb9q8qiLsFr0=vr0=vr0dc8meaabaqaciaacaGaaeqabaWaaeGaeaaakeaaimaacqWFZestaaa@3790@1, uses only dependencies to upstream positions, (*i*-), the second model, ℳ
 MathType@MTEF@5@5@+=feaafiart1ev1aaatCvAUfKttLearuWrP9MDH5MBPbIqV92AaeXatLxBI9gBamrtHrhAL1wy0L2yHvtyaeHbnfgDOvwBHrxAJfwnaebbnrfifHhDYfgasaacH8akY=wiFfYdH8Gipec8Eeeu0xXdbba9frFj0=OqFfea0dXdd9vqai=hGuQ8kuc9pgc9s8qqaq=dirpe0xb9q8qiLsFr0=vr0=vr0dc8meaabaqaciaacaGaaeqabaWaaeGaeaaakeaaimaacqWFZestaaa@3790@2, includes dependencies to upstream (*i*-), and downstream (*i*+) positions simultaneously, and the third model, ℳ
 MathType@MTEF@5@5@+=feaafiart1ev1aaatCvAUfKttLearuWrP9MDH5MBPbIqV92AaeXatLxBI9gBamrtHrhAL1wy0L2yHvtyaeHbnfgDOvwBHrxAJfwnaebbnrfifHhDYfgasaacH8akY=wiFfYdH8Gipec8Eeeu0xXdbba9frFj0=OqFfea0dXdd9vqai=hGuQ8kuc9pgc9s8qqaq=dirpe0xb9q8qiLsFr0=vr0=vr0dc8meaabaqaciaacaGaaeqabaWaaeGaeaaakeaaimaacqWFZestaaa@3790@3, incorporates only downstream (*i*+) dependencies. For each dependency model (ℳ
 MathType@MTEF@5@5@+=feaafiart1ev1aaatCvAUfKttLearuWrP9MDH5MBPbIqV92AaeXatLxBI9gBamrtHrhAL1wy0L2yHvtyaeHbnfgDOvwBHrxAJfwnaebbnrfifHhDYfgasaacH8akY=wiFfYdH8Gipec8Eeeu0xXdbba9frFj0=OqFfea0dXdd9vqai=hGuQ8kuc9pgc9s8qqaq=dirpe0xb9q8qiLsFr0=vr0=vr0dc8meaabaqaciaacaGaaeqabaWaaeGaeaaakeaaimaacqWFZestaaa@3790@1-ℳ
 MathType@MTEF@5@5@+=feaafiart1ev1aaatCvAUfKttLearuWrP9MDH5MBPbIqV92AaeXatLxBI9gBamrtHrhAL1wy0L2yHvtyaeHbnfgDOvwBHrxAJfwnaebbnrfifHhDYfgasaacH8akY=wiFfYdH8Gipec8Eeeu0xXdbba9frFj0=OqFfea0dXdd9vqai=hGuQ8kuc9pgc9s8qqaq=dirpe0xb9q8qiLsFr0=vr0=vr0dc8meaabaqaciaacaGaaeqabaWaaeGaeaaakeaaimaacqWFZestaaa@3790@3), the probability of observing an amino acid at a given position is defined using the dependence patterns selected from Table 7. For instance, according to the model ℳ
 MathType@MTEF@5@5@+=feaafiart1ev1aaatCvAUfKttLearuWrP9MDH5MBPbIqV92AaeXatLxBI9gBamrtHrhAL1wy0L2yHvtyaeHbnfgDOvwBHrxAJfwnaebbnrfifHhDYfgasaacH8akY=wiFfYdH8Gipec8Eeeu0xXdbba9frFj0=OqFfea0dXdd9vqai=hGuQ8kuc9pgc9s8qqaq=dirpe0xb9q8qiLsFr0=vr0=vr0dc8meaabaqaciaacaGaaeqabaWaaeGaeaaakeaaimaacqWFZestaaa@3790@2, the conditional probability of observing an amino acid at position *i *= *N*3 of an *α*-helix segment becomes P_N_3__ (R_i_l hi−23,hi−33,hi+23
 MathType@MTEF@5@5@+=feaafiart1ev1aaatCvAUfKttLearuWrP9MDH5MBPbIqV92AaeXatLxBI9gBaebbnrfifHhDYfgasaacH8akY=wiFfYdH8Gipec8Eeeu0xXdbba9frFj0=OqFfea0dXdd9vqai=hGuQ8kuc9pgc9s8qqaq=dirpe0xb9q8qiLsFr0=vr0=vr0dc8meaabaqaciaacaGaaeqabaqabeGadaaakeaacqWGObaAdaqhaaWcbaGaemyAaKMaeyOeI0IaeGOmaidabaGaeG4mamdaaOGaeiilaWIaemiAaG2aa0baaSqaaiabdMgaPjabgkHiTiabiodaZaqaaiabiodaZaaakiabcYcaSiabdIgaOnaaDaaaleaacqWGPbqAcqGHRaWkcqaIYaGmaeaacqaIZaWmaaaaaa@3F93@). By multiplying the conditional probabilities selected from Table 7 as formulated in Eq. 9, we obtain the propensity value for the observation of the amino acid segment under the specified model. In the case of ℳ
 MathType@MTEF@5@5@+=feaafiart1ev1aaatCvAUfKttLearuWrP9MDH5MBPbIqV92AaeXatLxBI9gBamrtHrhAL1wy0L2yHvtyaeHbnfgDOvwBHrxAJfwnaebbnrfifHhDYfgasaacH8akY=wiFfYdH8Gipec8Eeeu0xXdbba9frFj0=OqFfea0dXdd9vqai=hGuQ8kuc9pgc9s8qqaq=dirpe0xb9q8qiLsFr0=vr0=vr0dc8meaabaqaciaacaGaaeqabaWaaeGaeaaakeaaimaacqWFZestaaa@3790@1, and ℳ
 MathType@MTEF@5@5@+=feaafiart1ev1aaatCvAUfKttLearuWrP9MDH5MBPbIqV92AaeXatLxBI9gBamrtHrhAL1wy0L2yHvtyaeHbnfgDOvwBHrxAJfwnaebbnrfifHhDYfgasaacH8akY=wiFfYdH8Gipec8Eeeu0xXdbba9frFj0=OqFfea0dXdd9vqai=hGuQ8kuc9pgc9s8qqaq=dirpe0xb9q8qiLsFr0=vr0=vr0dc8meaabaqaciaacaGaaeqabaWaaeGaeaaakeaaimaacqWFZestaaa@3790@3, this product gives the segment likelihood expression, which is a properly normalized probability value *P*(R[Sj−1+1:Sj]
 MathType@MTEF@5@5@+=feaafiart1ev1aaatCvAUfKttLearuWrP9MDH5MBPbIqV92AaeXatLxBI9gBaebbnrfifHhDYfgasaacH8akY=wiFfYdH8Gipec8Eeeu0xXdbba9frFj0=OqFfea0dXdd9vqai=hGuQ8kuc9pgc9s8qqaq=dirpe0xb9q8qiLsFr0=vr0=vr0dc8meaabaqaciaacaGaaeqabaqabeGadaaakeaaieqacqWFsbGudaWgaaWcbaWaamWaaeaacqWGtbWudaWgaaadbaGaemOAaOMaeyOeI0IaeGymaedabeaaliabgUcaRiabigdaXiabcQda6iabdofatnaaBaaameaacqWGQbGAaeqaaaWccaGLBbGaayzxaaaabeaaaaa@3A30@ | **S**, **T**). Hence, ℳ
 MathType@MTEF@5@5@+=feaafiart1ev1aaatCvAUfKttLearuWrP9MDH5MBPbIqV92AaeXatLxBI9gBamrtHrhAL1wy0L2yHvtyaeHbnfgDOvwBHrxAJfwnaebbnrfifHhDYfgasaacH8akY=wiFfYdH8Gipec8Eeeu0xXdbba9frFj0=OqFfea0dXdd9vqai=hGuQ8kuc9pgc9s8qqaq=dirpe0xb9q8qiLsFr0=vr0=vr0dc8meaabaqaciaacaGaaeqabaWaaeGaeaaakeaaimaacqWFZestaaa@3790@1, and ℳ
 MathType@MTEF@5@5@+=feaafiart1ev1aaatCvAUfKttLearuWrP9MDH5MBPbIqV92AaeXatLxBI9gBamrtHrhAL1wy0L2yHvtyaeHbnfgDOvwBHrxAJfwnaebbnrfifHhDYfgasaacH8akY=wiFfYdH8Gipec8Eeeu0xXdbba9frFj0=OqFfea0dXdd9vqai=hGuQ8kuc9pgc9s8qqaq=dirpe0xb9q8qiLsFr0=vr0=vr0dc8meaabaqaciaacaGaaeqabaWaaeGaeaaakeaaimaacqWFZestaaa@3790@3 are probabilistic models. For the model ℳ
 MathType@MTEF@5@5@+=feaafiart1ev1aaatCvAUfKttLearuWrP9MDH5MBPbIqV92AaeXatLxBI9gBamrtHrhAL1wy0L2yHvtyaeHbnfgDOvwBHrxAJfwnaebbnrfifHhDYfgasaacH8akY=wiFfYdH8Gipec8Eeeu0xXdbba9frFj0=OqFfea0dXdd9vqai=hGuQ8kuc9pgc9s8qqaq=dirpe0xb9q8qiLsFr0=vr0=vr0dc8meaabaqaciaacaGaaeqabaWaaeGaeaaakeaaimaacqWFZestaaa@3790@2, we rather obtain a score *Q*(R[Sj−1+1:Sj]
 MathType@MTEF@5@5@+=feaafiart1ev1aaatCvAUfKttLearuWrP9MDH5MBPbIqV92AaeXatLxBI9gBaebbnrfifHhDYfgasaacH8akY=wiFfYdH8Gipec8Eeeu0xXdbba9frFj0=OqFfea0dXdd9vqai=hGuQ8kuc9pgc9s8qqaq=dirpe0xb9q8qiLsFr0=vr0=vr0dc8meaabaqaciaacaGaaeqabaqabeGadaaakeaaieqacqWFsbGudaWgaaWcbaWaamWaaeaacqWGtbWudaWgaaadbaGaemOAaOMaeyOeI0IaeGymaedabeaaliabgUcaRiabigdaXiabcQda6iabdofatnaaBaaameaacqWGQbGAaeqaaaWccaGLBbGaayzxaaaabeaaaaa@3A30@ | **S**, **T**) that represents the potential of a given amino acid segment to adopt a particular secondary structure conformation. This scoring system can be used to characterize amino acid segments in terms of their propensity to form structures of different types and when uniformly applied to compute segment potentials, allows to implement algorithms following the theory of hidden semi-Markov models. Implementing three different models enables to generate three predictions each specializing in a different section of the dependency structure. Those predictions can then be combined to get a final prediction sequence, as explained in the next section.

### The hidden semi-Markov model and computational methods

Amino acid and DNA sequences have been successfully analyzed using hidden Markov models (HMM) as the character strings generated in "left-to-right" direction. For a comprehensive introduction to HMMs, see [63].

**Figure 3:**
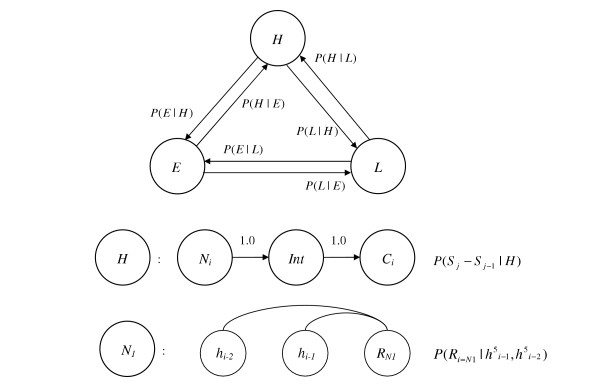
HSMM architecture. Transitions between secondary structure states are modeled as first order Markovian (top figure). Each state contains separate models for terminal and internal positions (middle figure). Position specific models have characteristic dependency structures with conditional independence of the amino acids (i.g. bottom figure shows dependency diagram for the *N*_1 _residue of a structural segment under the model *M*_1_).

Here, we consider a hidden semi-Markov model (HSMM) also known as HMM with duration. Such type of model was earlier used in gene finding methods, such as Genie [64], GenScan [65] and GeneMark.hmm [66]. The HSMM technique was introduced for protein structure prediction by Schmidler *et al*. [14]. In a HSMM, a transition from a hidden state into itself cannot occur, while a hidden state can emit a whole string of symbols rather than a single symbol. The hidden states of the model used in protein secondary structure prediction are the structural states {H, E, L} designating *α*-helix, *β*-strand and loop segments, respectively. The state transitions occur with probabilities *P*(*T*_*j *_| *T*_*j*-1_) thus forming a first order Markov chain. At each hidden state, an amino acid segment with uniform structure is generated according to a given length distribution *P*(*S*_*j *_| *S*_*j*-1_, *T*_*j*_), and the likelihood *P*(R[Sj−1+1:Sj]
 MathType@MTEF@5@5@+=feaafiart1ev1aaatCvAUfKttLearuWrP9MDH5MBPbIqV92AaeXatLxBI9gBaebbnrfifHhDYfgasaacH8akY=wiFfYdH8Gipec8Eeeu0xXdbba9frFj0=OqFfea0dXdd9vqai=hGuQ8kuc9pgc9s8qqaq=dirpe0xb9q8qiLsFr0=vr0=vr0dc8meaabaqaciaacaGaaeqabaqabeGadaaakeaaieqacqWFsbGudaWgaaWcbaWaamWaaeaacqWGtbWudaWgaaadbaGaemOAaOMaeyOeI0IaeGymaedabeaaliabgUcaRiabigdaXiabcQda6iabdofatnaaBaaameaacqWGQbGAaeqaaaWccaGLBbGaayzxaaaabeaaaaa@3A30@ | *S*_*j*-1_, *S*_*j*_, *T*_*j*_) (Fig. 3).

Having defined this HSMM, we can consider the protein secondary structure prediction problem as the problem of finding the sequence of hidden states with the highest *a posteriori *probability given the amino acid sequence. One efficient algorithm to solve this optimization problem is well known. Given an amino acid sequence **R**, the vector (**S**, **T**)* = arg max *P*(**S**, **T **| **R**) can be found using the Viterbi algorithm. Here lies a subtle difference between the result that can be delivered by the Viterbi algorithm and the result needed in the traditional statement of the protein secondary structure prediction problem.

The Viterbi path does not directly optimize the three-state-per residue accuracy (*Q*_3_):

Q3=Total # of correctly predicted structural statesTotal # of observed amino acids.     (10)
 MathType@MTEF@5@5@+=feaafiart1ev1aaatCvAUfKttLearuWrP9MDH5MBPbIqV92AaeXatLxBI9gBaebbnrfifHhDYfgasaacH8akY=wiFfYdH8Gipec8Eeeu0xXdbba9frFj0=OqFfea0dXdd9vqai=hGuQ8kuc9pgc9s8qqaq=dirpe0xb9q8qiLsFr0=vr0=vr0dc8meaabaqaciaacaGaaeqabaqabeGadaaakeaacqWGrbqudaWgaaWcbaGaeG4mamdabeaakiabg2da9maalaaabaGaeeivaqLaee4Ba8MaeeiDaqNaeeyyaeMaeeiBaWMaeeiiaaIaee4iamIaeeiiaaIaee4Ba8MaeeOzayMaeeiiaaIaee4yamMaee4Ba8MaeeOCaiNaeeOCaiNaeeyzauMaee4yamMaeeiDaqNaeeiBaWMaeeyEaKNaeeiiaaIaeeiCaaNaeeOCaiNaeeyzauMaeeizaqMaeeyAaKMaee4yamMaeeiDaqNaeeyzauMaeeizaqMaeeiiaaIaee4CamNaeeiDaqNaeeOCaiNaeeyDauNaee4yamMaeeiDaqNaeeyDauNaeeOCaiNaeeyyaeMaeeiBaWMaeeiiaaIaee4CamNaeeiDaqNaeeyyaeMaeeiDaqNaeeyzauMaee4CamhabaGaeeivaqLaee4Ba8MaeeiDaqNaeeyyaeMaeeiBaWMaeeiiaaIaee4iamIaeeiiaaIaee4Ba8MaeeOzayMaeeiiaaIaee4Ba8MaeeOyaiMaee4CamNaeeyzauMaeeOCaiNaeeODayNaeeyzauMaeeizaqMaeeiiaaIaeeyyaeMaeeyBa0MaeeyAaKMaeeOBa4Maee4Ba8MaeeiiaaIaeeyyaeMaee4yamMaeeyAaKMaeeizaqMaee4Camhaaiabc6caUiaaxMaacaWLjaWaaeWaaeaacqaIXaqmcqaIWaamaiaawIcacaGLPaaaaaa@99D3@

Also, the Viterbi algorithm might generate many different segmentations, which might have significant probability mass but are not optimal [14]. As an alternative to the Viterbi algorithm, we can determine the sequence of structural states that are most likely to occur in each position. This approach will use forward and backward algorithms (posterior decoding) generalized for HSMM [63]. Although the prediction sequence obtained by forward and backward algorithms might not be a perfectly valid state sequence (i.e. it might not be realized given the parameters of HSMM), the prediction measure defined as the marginal posterior probability distribution (Eq. 14) correlates very strongly with the prediction accuracy (*Q*_3_) [14]. The performance of the Viterbi and forward-backward algorithms are compared in Schmidler *et al*. [14]. Here, forward and backward variables are defined as follows (*n *is the total number of amino acids in a sequence):

αθ(j,t)=Pθ(R[1:j],S=j,T=t)=∑v=1j−1∑l∈SSαθ(v,l)Pθ(R[v+1:j]|Sprev=v,S=j,T=t)×Pθ(S=j|T=t,Sprev=v)Pθ(T=t|Tprev=l)αθ(1,t)=Pθ(R[1]|T=t,S=1)Pθ(S=1|T=t)Pθ(T=t)j=1,…,n     (11)
 MathType@MTEF@5@5@+=feaafiart1ev1aaatCvAUfKttLearuWrP9MDH5MBPbIqV92AaeXatLxBI9gBaebbnrfifHhDYfgasaacH8akY=wiFfYdH8Gipec8Eeeu0xXdbba9frFj0=OqFfea0dXdd9vqai=hGuQ8kuc9pgc9s8qqaq=dirpe0xb9q8qiLsFr0=vr0=vr0dc8meaabaqaciaacaGaaeqabaqabeGadaaakeaafaqadeqbbaaaaeaaiiGacqWFXoqydaahaaWcbeqaaiab=H7aXbaakmaabmaabaGaemOAaOMaeiilaWIaemiDaqhacaGLOaGaayzkaaGaeyypa0Jaemiuaa1aaWbaaSqabeaacqWF4oqCaaGcdaqadaqaaiabdkfasnaaBaaaleaadaWadaqaaiabigdaXiabcQda6iabdQgaQbGaay5waiaaw2faaaqabaGccqGGSaalcqWGtbWucqGH9aqpcqWGQbGAcqGGSaalcqWGubavcqGH9aqpcqWG0baDaiaawIcacaGLPaaaaeaaiiaacqGF9aqpdaaeWbqaamaaqafabaGae8xSde2aaWbaaSqabeaacqWF4oqCaaGcdaqadaqaaiabdAha2jabcYcaSiabdYgaSbGaayjkaiaawMcaaiabdcfaqnaaCaaaleqabaGae8hUdehaaOWaaeWaaeaacqWGsbGudaWgaaWcbaWaamWaaeaacqWG2bGDcqGHRaWkcqaIXaqmcqGG6aGocqWGQbGAaiaawUfacaGLDbaaaeqaaOGaeiiFaWNaem4uam1aaSbaaSqaaiabdchaWjabdkhaYjabdwgaLjabdAha2bqabaGccqGH9aqpcqWG2bGDcqGGSaalcqWGtbWucqGH9aqpcqWGQbGAcqGGSaalcqWGubavcqGH9aqpcqWG0baDaiaawIcacaGLPaaaaSqaaiabdYgaSjabgIGiolabdofatjabdofatbqab0GaeyyeIuoaaSqaaiabdAha2jabg2da9iabigdaXaqaaiabdQgaQjabgkHiTiabigdaXaqdcqGHris5aaGcbaGaey41aqRaemiuaa1aaWbaaSqabeaacqWF4oqCaaGcdaqadaqaaiabdofatjabg2da9iabdQgaQjabcYha8jabdsfaujabg2da9iabdsha0jabcYcaSiabdofatnaaBaaaleaacqWGWbaCcqWGYbGCcqWGLbqzcqWG2bGDaeqaaOGaeyypa0JaemODayhacaGLOaGaayzkaaGaemiuaa1aaWbaaSqabeaacqWF4oqCaaGcdaqadaqaaiabdsfaujabg2da9iabdsha0jabcYha8jabdsfaunaaBaaaleaacqWGWbaCcqWGYbGCcqWGLbqzcqWG2bGDaeqaaOGaeyypa0JaemiBaWgacaGLOaGaayzkaaaabaGae8xSde2aaWbaaSqabeaacqWF4oqCaaGcdaqadaqaaiabigdaXiabcYcaSiabdsha0bGaayjkaiaawMcaaiabg2da9iabdcfaqnaaCaaaleqabaGae8hUdehaaOWaaeWaaeaacqWGsbGudaWgaaWcbaWaamWaaeaacqaIXaqmaiaawUfacaGLDbaaaeqaaOGaeiiFaWNaemivaqLaeyypa0JaemiDaqNaeiilaWIaem4uamLaeyypa0JaeGymaedacaGLOaGaayzkaaGaemiuaa1aaWbaaSqabeaacqWF4oqCaaGcdaqadaqaaiabdofatjabg2da9iabigdaXiabcYha8jabdsfaujabg2da9iabdsha0bGaayjkaiaawMcaaiabdcfaqnaaCaaaleqabaGae8hUdehaaOWaaeWaaeaacqWGubavcqGH9aqpcqWG0baDaiaawIcacaGLPaaaaeaacqWGQbGAcqGH9aqpcqaIXaqmcqGGSaalcqWIMaYscqGGSaalcqWGUbGBaaGaaCzcaiaaxMaadaqadaqaaiabigdaXiabigdaXaGaayjkaiaawMcaaaaa@F02A@

βθ(j,t)=Pθ(R[j+1:n]|S=j,T=t)=∑v=j+1n∑l∈SSβθ(v,l)Pθ(R[j+1:v]|Sprev=v,S=j,Tnext=l)×Pθ(Snext=v|S=j,Tnext=l)Pθ(Tnext=l|T=t)βθ(n,t)=1j=n,…,1     (12)
 MathType@MTEF@5@5@+=feaafiart1ev1aaatCvAUfKttLearuWrP9MDH5MBPbIqV92AaeXatLxBI9gBaebbnrfifHhDYfgasaacH8akY=wiFfYdH8Gipec8Eeeu0xXdbba9frFj0=OqFfea0dXdd9vqai=hGuQ8kuc9pgc9s8qqaq=dirpe0xb9q8qiLsFr0=vr0=vr0dc8meaabaqaciaacaGaaeqabaqabeGadaaakeaafaqadeqbbaaaaeaaiiGacqWFYoGydaahaaWcbeqaaiab=H7aXbaakmaabmaabaGaemOAaOMaeiilaWIaemiDaqhacaGLOaGaayzkaaGaeyypa0Jaemiuaa1aaWbaaSqabeaacqWF4oqCaaGcdaqadaqaaiabdkfasnaaBaaaleaadaWadaqaaiabdQgaQjabgUcaRiabigdaXiabcQda6iabd6gaUbGaay5waiaaw2faaaqabaGccqGG8baFcqWGtbWucqGH9aqpcqWGQbGAcqGGSaalcqWGubavcqGH9aqpcqWG0baDaiaawIcacaGLPaaaaeaaiiaacqGF9aqpdaaeWbqaamaaqafabaGae8NSdi2aaWbaaSqabeaacqWF4oqCaaGcdaqadaqaaiabdAha2jabcYcaSiabdYgaSbGaayjkaiaawMcaaiabdcfaqnaaCaaaleqabaGae8hUdehaaOWaaeWaaeaacqWGsbGudaWgaaWcbaWaamWaaeaacqWGQbGAcqGHRaWkcqaIXaqmcqGG6aGocqWG2bGDaiaawUfacaGLDbaaaeqaaOGaeiiFaWNaem4uam1aaSbaaSqaaiabdchaWjabdkhaYjabdwgaLjabdAha2bqabaGccqGH9aqpcqWG2bGDcqGGSaalcqWGtbWucqGH9aqpcqWGQbGAcqGGSaalcqWGubavdaWgaaWcbaGaemOBa4MaemyzauMaemiEaGNaemiDaqhabeaakiabg2da9iabdYgaSbGaayjkaiaawMcaaaWcbaGaemiBaWMaeyicI4Saem4uamLaem4uamfabeqdcqGHris5aaWcbaGaemODayNaeyypa0JaemOAaOMaey4kaSIaeGymaedabaGaemOBa4ganiabggHiLdaakeaacqGHxdaTcqWGqbaudaahaaWcbeqaaiab=H7aXbaakmaabmaabaGaem4uam1aaSbaaSqaaiabd6gaUjabdwgaLjabdIha4jabdsha0bqabaGccqGH9aqpcqWG2bGDcqGG8baFcqWGtbWucqGH9aqpcqWGQbGAcqGGSaalcqWGubavdaWgaaWcbaGaemOBa4MaemyzauMaemiEaGNaemiDaqhabeaakiabg2da9iabdYgaSbGaayjkaiaawMcaaiabdcfaqnaaCaaaleqabaGae8hUdehaaOWaaeWaaeaacqWGubavdaWgaaWcbaGaemOBa4MaemyzauMaemiEaGNaemiDaqhabeaakiabg2da9iabdYgaSjabcYha8jabdsfaujabg2da9iabdsha0bGaayjkaiaawMcaaaqaaiab=j7aInaaCaaaleqabaGae8hUdehaaOWaaeWaaeaacqWGUbGBcqGGSaalcqWG0baDaiaawIcacaGLPaaacqGH9aqpcqaIXaqmaeaacqWGQbGAcqGH9aqpcqWGUbGBcqGGSaalcqWIMaYscqGGSaalcqaIXaqmaaGaaCzcaiaaxMaadaqadaqaaiabigdaXiabikdaYaGaayjkaiaawMcaaaaa@D94B@

The forward variable *α*^*θ*^(*j*, *t*) is the joint probability of observing the amino acid sequence up to position *j*, and a secondary structure segment that ends at position *j *with type *t*.  Here, *θ *represents the statistical dependency model. Similarly, the backward variable *β*^*θ*^(*j*, *t*) defines the conditional probability of observing the amino acid sequence in positions *j *+ 1 to *n*, and a secondary structure segment that ends at position *j *with type *t*. Then, the *a posteriori *probability for a hidden state in position *i *to be either an *α*-helix, *β*-strand or loop is computed via all possible segmentations that include position *i *(Eq. 13).  The hidden state at position *i *is identified as the state with maximum *a posteriori *probability. Finally, the whole predicted sequence of hidden states is defined by Eq.14.

Pθ(TRi|R)=∑j=1i−1∑k=in∑l∈SSαθ(j,l)βθ(k,t)Pθ(T=t|Tprev=l)×Pθ(S=k|Sprev=j,T=t)×pθ(R[j+1:k]|Sprev=j,S=k,T=t)/Pθ(R)     (13)
 MathType@MTEF@5@5@+=feaafiart1ev1aaatCvAUfKttLearuWrP9MDH5MBPbIqV92AaeXatLxBI9gBaebbnrfifHhDYfgasaacH8akY=wiFfYdH8Gipec8Eeeu0xXdbba9frFj0=OqFfea0dXdd9vqai=hGuQ8kuc9pgc9s8qqaq=dirpe0xb9q8qiLsFr0=vr0=vr0dc8meaabaqaciaacaGaaeqabaqabeGadaaakeaafaqadeWabaaabaGaemiuaa1aaWbaaSqabeaaiiGacqWF4oqCaaGcdaqadaqaaiabdsfaunaaBaaaleaacqWGsbGudaWgaaadbaGaemyAaKgabeaaaSqabaGccqGG8baFcqWGsbGuaiaawIcacaGLPaaacqGH9aqpdaaeWbqaamaaqahabaWaaabuaeaacqWFXoqydaahaaWcbeqaaiab=H7aXbaakmaabmaabaGaemOAaOMaeiilaWIaemiBaWgacaGLOaGaayzkaaaaleaacqWGSbaBcqGHiiIZcqWGtbWucqWGtbWuaeqaniabggHiLdGccqWFYoGydaahaaWcbeqaaiab=H7aXbaakmaabmaabaGaem4AaSMaeiilaWIaemiDaqhacaGLOaGaayzkaaaaleaacqWGRbWAcqGH9aqpcqWGPbqAaeaacqWGUbGBa0GaeyyeIuoakiabdcfaqnaaCaaaleqabaGae8hUdehaaOWaaeWaaeaacqWGubavcqGH9aqpcqWG0baDcqGG8baFcqWGubavdaWgaaWcbaGaemiCaaNaemOCaiNaemyzauMaemODayhabeaakiabg2da9iabdYgaSbGaayjkaiaawMcaaaWcbaGaemOAaOMaeyypa0JaeGymaedabaGaemyAaKMaeyOeI0IaeGymaedaniabggHiLdaakeaacqGHxdaTcqWGqbaudaahaaWcbeqaaiab=H7aXbaakmaabmaabaGaem4uamLaeyypa0Jaem4AaSMaeiiFaWNaem4uam1aaSbaaSqaaiabdchaWjabdkhaYjabdwgaLjabdAha2bqabaGccqGH9aqpcqWGQbGAcqGGSaalcqWGubavcqGH9aqpcqWG0baDaiaawIcacaGLPaaaaeaacqGHxdaTcqWGWbaCdaahaaWcbeqaaiab=H7aXbaakmaabmaabaGaemOuai1aaSbaaSqaamaadmaabaGaemOAaOMaey4kaSIaeGymaeJaeiOoaOJaem4AaSgacaGLBbGaayzxaaaabeaakiabcYha8jabdofatnaaBaaaleaacqWGWbaCcqWGYbGCcqWGLbqzcqWG2bGDaeqaaOGaeyypa0JaemOAaOMaeiilaWIaem4uamLaeyypa0Jaem4AaSMaeiilaWIaemivaqLaeyypa0JaemiDaqhacaGLOaGaayzkaaGaei4la8Iaemiuaa1aaWbaaSqabeaacqWF4oqCaaGcdaqadaqaaiabdkfasbGaayjkaiaawMcaaaaacaWLjaGaaCzcamaabmaabaGaeGymaeJaeG4mamdacaGLOaGaayzkaaaaaa@BE4C@

(S,T)*=arg⁡max⁡(S,T){Pθ(TRi|R)}i=1n     (14)
 MathType@MTEF@5@5@+=feaafiart1ev1aaatCvAUfKttLearuWrP9MDH5MBPbIqV92AaeXatLxBI9gBaebbnrfifHhDYfgasaacH8akY=wiFfYdH8Gipec8Eeeu0xXdbba9frFj0=OqFfea0dXdd9vqai=hGuQ8kuc9pgc9s8qqaq=dirpe0xb9q8qiLsFr0=vr0=vr0dc8meaabaqaciaacaGaaeqabaqabeGadaaakeaadaqadaqaaiabdofatjabcYcaSiabdsfaubGaayjkaiaawMcaamaaCaaaleqabaGaeiOkaOcaaOGaeyypa0JagiyyaeMaeiOCaiNaei4zaC2aaCbeaeaacyGGTbqBcqGGHbqycqGG4baEaSqaaiabcIcaOiabdofatjabcYcaSiabdsfaujabcMcaPaqabaGcdaGadaqaaiabdcfaqnaaCaaaleqabaacciGae8hUdehaaOWaaeWaaeaacqWGubavdaWgaaWcbaGaemOuai1aaSbaaWqaaiabdMgaPbqabaaaleqaaOGaeiiFaWNaemOuaifacaGLOaGaayzkaaaacaGL7bGaayzFaaWaa0baaSqaaiabdMgaPjabg2da9iabigdaXaqaaiabd6gaUbaakiaaxMaacaWLjaWaaeWaaeaacqaIXaqmcqaI0aanaiaawIcacaGLPaaaaaa@5842@

The computational complexity of this algorithm is *O*(*n*^3^). If the maximum size of a segment is limited by a value D, the first summation in Eq. 11 starts at (*j *- *D*), and the first summation in Eq. 12 ends at (*j *+ *D*) reducing the computational cost to *O*(*nD*^2^).

Note that, forward and backward variables are computed by multiplying probabilities, which are less than 1, and as the sequence gets longer, these variables approximate to zero after a certain position. Hence it is necessary to introduce a scaling procedure to prevent numerical underflow. The scaling for a "classic" HMM is described in [63]. This procedure can easily be generalized for an HSMM, where the scaling coefficients are introduced at every *D *positions.

This completes the derivation of the algorithm for a single model. Since we are utilizing three dependency models, *θ *= ℳ
 MathType@MTEF@5@5@+=feaafiart1ev1aaatCvAUfKttLearuWrP9MDH5MBPbIqV92AaeXatLxBI9gBamrtHrhAL1wy0L2yHvtyaeHbnfgDOvwBHrxAJfwnaebbnrfifHhDYfgasaacH8akY=wiFfYdH8Gipec8Eeeu0xXdbba9frFj0=OqFfea0dXdd9vqai=hGuQ8kuc9pgc9s8qqaq=dirpe0xb9q8qiLsFr0=vr0=vr0dc8meaabaqaciaacaGaaeqabaWaaeGaeaaakeaaimaacqWFZestaaa@3790@1, ℳ
 MathType@MTEF@5@5@+=feaafiart1ev1aaatCvAUfKttLearuWrP9MDH5MBPbIqV92AaeXatLxBI9gBamrtHrhAL1wy0L2yHvtyaeHbnfgDOvwBHrxAJfwnaebbnrfifHhDYfgasaacH8akY=wiFfYdH8Gipec8Eeeu0xXdbba9frFj0=OqFfea0dXdd9vqai=hGuQ8kuc9pgc9s8qqaq=dirpe0xb9q8qiLsFr0=vr0=vr0dc8meaabaqaciaacaGaaeqabaWaaeGaeaaakeaaimaacqWFZestaaa@3790@2, ℳ
 MathType@MTEF@5@5@+=feaafiart1ev1aaatCvAUfKttLearuWrP9MDH5MBPbIqV92AaeXatLxBI9gBamrtHrhAL1wy0L2yHvtyaeHbnfgDOvwBHrxAJfwnaebbnrfifHhDYfgasaacH8akY=wiFfYdH8Gipec8Eeeu0xXdbba9frFj0=OqFfea0dXdd9vqai=hGuQ8kuc9pgc9s8qqaq=dirpe0xb9q8qiLsFr0=vr0=vr0dc8meaabaqaciaacaGaaeqabaWaaeGaeaaakeaaimaacqWFZestaaa@3790@3, it becomes necessary to combine the outputs of the three models with an appropriate function. In our simulations, we implemented averaging and maximum functions to perform this task and observed that the averaging function gives a better performance. The final prediction sequence is then computed as:

PC(TRi|R)=(Pℳ1(TRi|R)+Pℳ2(TRi|R)+Pℳ3(TRi|R))/3(S,T)*=arg⁡max⁡(S,T){PC(TRi|R)}i=1n     (15)
 MathType@MTEF@5@5@+=feaafiart1ev1aaatCvAUfKttLearuWrP9MDH5MBPbIqV92AaeXatLxBI9gBamrtHrhAL1wy0L2yHvtyaeHbnfgDOvwBHrxAJfwnaebbnrfifHhDYfgasaacH8akY=wiFfYdH8Gipec8Eeeu0xXdbba9frFj0=OqFfea0dXdd9vqai=hGuQ8kuc9pgc9s8qqaq=dirpe0xb9q8qiLsFr0=vr0=vr0dc8meaabaqaciaacaGaaeqabaWaaeGaeaaakeaafaqabeGadaaabaGaemiuaa1aaWbaaSqabeaacqWGdbWqaaGccqGGOaakcqWGubavdaWgaaWcbaGaemOuai1aaSbaaWqaaiabdMgaPbqabaaaleqaaOGaeiiFaWNaemOuaiLaeiykaKcabaGaeyypa0dabaGaeiikaGIaemiuaa1aaWbaaSqabeaaimaacqWFZestieaacqGFXaqmaaGccqGGOaakcqWGubavdaWgaaWcbaGaemOuai1aaSbaaWqaaiabdMgaPbqabaaaleqaaOGaeiiFaWNaemOuaiLaeiykaKIaey4kaSIaemiuaa1aaWbaaSqabeaacqWFZestcqGFYaGmaaGccqGGOaakcqWGubavdaWgaaWcbaGaemOuai1aaSbaaWqaaiabdMgaPbqabaaaleqaaOGaeiiFaWNaemOuaiLaeiykaKIaey4kaSIaemiuaa1aaWbaaSqabeaacqWFZestcqGFZaWmaaGccqGGOaakcqWGubavdaWgaaWcbaGaemOuai1aaSbaaWqaaiabdMgaPbqabaaaleqaaOGaeiiFaWNaemOuaiLaeiykaKIaeiykaKIaei4la8IaeG4mamdabaGaeiikaGIaem4uamLaeiilaWIaemivaqLaeiykaKYaaWbaaSqabeaacqGGQaGkaaaakeaacqGH9aqpaeaacyGGHbqycqGGYbGCcqGGNbWzdaWfqaqaaiGbc2gaTjabcggaHjabcIha4bWcbaGaeiikaGIaem4uamLaeiilaWIaemivaqLaeiykaKcabeaakmaacmaabaGaemiuaa1aaWbaaSqabeaacqWGdbWqaaGccqGGOaakcqWGubavdaWgaaWcbaGaemOuai1aaSbaaWqaaiabdMgaPbqabaaaleqaaOGaeiiFaWNaemOuaiLaeiykaKcacaGL7bGaayzFaaWaa0baaSqaaiabdMgaPjabg2da9iabigdaXaqaaiabd6gaUbaaaaGccaWLjaGaaCzcamaabmaabaGaeGymaeJaeGynaudacaGLOaGaayzkaaaaaa@96E9@

### Iterative model training

**Figure 4:**
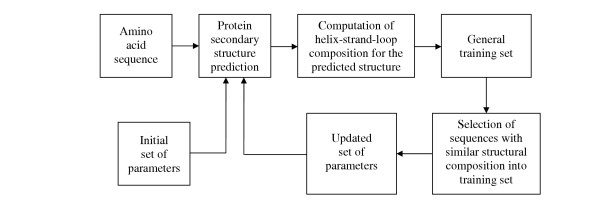
Iterative training method diagram. Initial set of model parameters is precomputed from the general training set.

To improve the estimation of the model parameters, we implemented an iterative training procedure. Upon obtaining an initial secondary structure prediction for a given amino acid sequence, we re-adjust the HSMM parameters using proteins that have similar structural features and repeat the prediction step. That is, once we obtain the prediction result for a test sequence, we compute the *α*-helix, *β*-strand, and loop compositions (the percentages of *α*-helix, *β*-strand, and loop predictions). We then remove from the training set those sequences that do not have a similar secondary structure composition. To assess the similarity between the prediction sequence and a training set protein, we compute the absolute value differences of the composition values and apply a fixed threshold. If the differences for all secondary structure types are less than the threshold, then the two sequences are assumed to be similar. The dataset reduction step is followed by the re-estimation of the HSMM parameters and the prediction of the secondary structure (Fig. 4). Note that, in the second and all subsequent iterations, we always start from the initial data set of training sequences and use the predicted sequence to reconstruct the training set. This approach prevents the iterations from sidetracking and converging to an incorrect result.

Although affinity in structural composition does not guarantee structural similarity, using this measure allows us to reduce the training set to proteins that belong to more closely related SCOP families [67]. Thus, for example, a prediction of a structure from an all-*α *class is likely to be followed by a training using proteins having high *α*-helix content. In our simulations, we observed that after several iterations (no more than 3) the predicted secondary structure sequence did not change indicating the algorithm convergence.

## Authors' contributions

MB and YA conceived and coordinated the study. MB introduced the ideas on the statistical analysis and the iterative training method. ZA introduced the dependency models and further developed the iterative training method. ZA implemented the statistical analysis, prediction algorithms and evaluated their performance. ZA and MB did the editing before submission.

## Supplementary Material

Additional File 1 Segment Overlap Score. In this file, the performances of the methods BSPSS and IPSSP are evaluated and compared on the Segment Overlap (SOV) measure, which is based on the average overlap between the observed and the predicted segments. Click here for file

Additional File 2 Reliability Measures. In this file, the performances of the methods BSPSS and IPSSP are evaluated and compared on two reliability measures: the prediction confidence and the percentage of predicted positions. Both measures are computed with respect to the prediction threshold. Click here for file

## Figures and Tables

**Table 1:** Number of proteins with known functional domains.

	# Proteins	# Proteins with Pfam hit	(%)
Bacteria	623,037	450,962	72.38
Archaea	50,406	33,259	65.98
Eukaryota	284,392	187,472	65.92
Total	957,835	671,693	70.13

**Table 2:** Statistics of hypothetical proteins and orphan proteins observed in the recently sequenced genomes (year 2004).

	# Proteins	(%) hypothetical proteins	(%) orphans in hypotheticals
Sulfolobus islandicus (Archaea)	197	65.98	57.69
Bacillus claussi (Bacteria)	4121	31.64	18.66
Gallus gallus (Eukaryota)	29,172	11.84	32.4

**Table 3:** The matrix of transition probabilities, *P*(*T*_*j *_| *T*_*j*-1_), used in the hidden semi-Markov model. Rows represent *T*_*j*-1 _values.

*P*(*T*_*j *_| *T*_*j*-1_)	H	E	L
H	-	0.031	0.969
E	0.029	-	0.971
L	0.314	0.686	-

**Table 4:** Correlations at the amino acid level as characterized by the *χ*^2 ^measure (PDB_SELECT set).

	Helix		Strand		Loop	
Separation	*χ*^2^	# of pairs	*χ*^2^	# of pairs	*χ*^2^	# of pairs

1	1854.34	118,324	**2579.85**	60,423	**9600.85**	154,404
2	**7008.83**	103,853	**1832.78**	44,121	5774.58	124,249
3	**2454.03**	89,414	1116.65	30,909	4828.13	100,325
4	**5095.27**	77,302	535.02	20,336	2276.21	80,930
5	2052.68	67,036	461.70	12,584	1298.16	66,109
6	1295.46	57,602	398.44	7361	950.66	54,993
7	2196.94	49,017	392.93	4196	895.42	46,391
8	627.00	41,350	355.81	2292	761.48	39,611

**Table 5:** KL distance between distributions of amino acids in proximal and internal positions (PDB_SELECT set).

KL-dis	N1	N2	N3	N4	C4	C3	C2	C1
*α*-Helix	**0.402**	**0.194**	**0.100**	**0.053**	0.018	0.018	0.020	**0.036**
*β*-Strand	**0.047**	**0.025**	0.019	-	-	0.021	**0.039**	**0.074**
Loop	**0.045**	0.019	0.008	0.003	0.004	0.008	**0.026**	**0.028**

**Table 6:** Position specific correlations as characterized by the *χ*^2 ^measure in *α*-helix proximal positions (PDB_SELECT set).

*χ*^2^	*i *- 5	*i *- 4	*i *- 3	*i *- 2	*i *- 1	*i *+ 1	*i *+ 2	*i *+ 3	*i *+ 4	*i *+ 5
N1	380.33	491.35	416.40	524.46	**708.76**	**770.43**	**982.18**	**875.59**	**1132.54**	487.41
N2	410.29	409.30	637.47	**1029.24**	**770.43**	**805.38**	**993.92**	619.44	**872.68**	594.32
N3	421.77	591.87	**2000.33**	**731.30**	661.04	**702.25**	**844.97**	697.18	694.83	652.90
N4	538.17	482.28	552.22	**649.11**	614.21	470.58	**827.26**	465.17	**1055.63**	468.99
C4	604.79	**830.18**	696.89	**1082.25**	481.05	463.31	**933.17**	578.54	657.20	527.83
C3	628.98	**963.03**	632.99	**1181.51**	497.00	527.86	**903.81**	485.95	443.62	370.97
C2	549.77	**1261.04**	624.90	**1270.42**	603.44	**717.04**	591.22	507.21	397.44	378.79
C1	563.37	**1213.12**	**714.48**	**1300.46**	**810.35**	**1266.49**	631.45	482.92	476.19	454.61

**Table 7:** Positional dependencies within structural segments for the models ℳ
 MathType@MTEF@5@5@+=feaafiart1ev1aaatCvAUfKttLearuWrP9MDH5MBPbIqV92AaeXatLxBI9gBamrtHrhAL1wy0L2yHvtyaeHbnfgDOvwBHrxAJfwnaebbnrfifHhDYfgasaacH8akY=wiFfYdH8Gipec8Eeeu0xXdbba9frFj0=OqFfea0dXdd9vqai=hGuQ8kuc9pgc9s8qqaq=dirpe0xb9q8qiLsFr0=vr0=vr0dc8meaabaqaciaacaGaaeqabaWaaeGaeaaakeaaimaacqWFZestaaa@3790@1, ℳ
 MathType@MTEF@5@5@+=feaafiart1ev1aaatCvAUfKttLearuWrP9MDH5MBPbIqV92AaeXatLxBI9gBamrtHrhAL1wy0L2yHvtyaeHbnfgDOvwBHrxAJfwnaebbnrfifHhDYfgasaacH8akY=wiFfYdH8Gipec8Eeeu0xXdbba9frFj0=OqFfea0dXdd9vqai=hGuQ8kuc9pgc9s8qqaq=dirpe0xb9q8qiLsFr0=vr0=vr0dc8meaabaqaciaacaGaaeqabaWaaeGaeaaakeaaimaacqWFZestaaa@3790@2, and ℳ
 MathType@MTEF@5@5@+=feaafiart1ev1aaatCvAUfKttLearuWrP9MDH5MBPbIqV92AaeXatLxBI9gBamrtHrhAL1wy0L2yHvtyaeHbnfgDOvwBHrxAJfwnaebbnrfifHhDYfgasaacH8akY=wiFfYdH8Gipec8Eeeu0xXdbba9frFj0=OqFfea0dXdd9vqai=hGuQ8kuc9pgc9s8qqaq=dirpe0xb9q8qiLsFr0=vr0=vr0dc8meaabaqaciaacaGaaeqabaWaaeGaeaaakeaaimaacqWFZestaaa@3790@3. hj3
 MathType@MTEF@5@5@+=feaafiart1ev1aaatCvAUfKttLearuWrP9MDH5MBPbIqV92AaeXatLxBI9gBaebbnrfifHhDYfgasaacH8akY=wiFfYdH8Gipec8Eeeu0xXdbba9frFj0=OqFfea0dXdd9vqai=hGuQ8kuc9pgc9s8qqaq=dirpe0xb9q8qiLsFr0=vr0=vr0dc8meaabaqaciaacaGaaeqabaqabeGadaaakeaacqWGObaAdaqhaaWcbaGaemOAaOgabaGaeG4mamdaaaaa@3083@ ∈ {*hydrophobic*, *neutral*, *hydrohilic*} indicates the hydrophobicity class of the amino acid *R*_*j*_, where hydrophobic = {*A*, *M*, *C*, *F*, *L*, *V*, *I*}, neutral = {*P*, *Y*, *W*, *S*, *T*, *G*}, hydrophilic = {*R*, *K*, *N*, *D*, *Q*, *E*, *H*}. hj5
 MathType@MTEF@5@5@+=feaafiart1ev1aaatCvAUfKttLearuWrP9MDH5MBPbIqV92AaeXatLxBI9gBaebbnrfifHhDYfgasaacH8akY=wiFfYdH8Gipec8Eeeu0xXdbba9frFj0=OqFfea0dXdd9vqai=hGuQ8kuc9pgc9s8qqaq=dirpe0xb9q8qiLsFr0=vr0=vr0dc8meaabaqaciaacaGaaeqabaqabeGadaaakeaacqWGObaAdaqhaaWcbaGaemOAaOgabaGaeGynaudaaaaa@3087@ is a 5 letter alphabet with groups defined as {*P*, *G*}, {*E*, *K*, *R*, *Q*}, {*D*, *S*, *N*, *T*, *H*, *C*}, {*I*, *V*, *W*, *Y*, *F*}, {*A*, *L*, *M*}.

		ℳ MathType@MTEF@5@5@+=feaafiart1ev1aaatCvAUfKttLearuWrP9MDH5MBPbIqV92AaeXatLxBI9gBamrtHrhAL1wy0L2yHvtyaeHbnfgDOvwBHrxAJfwnaebbnrfifHhDYfgasaacH8akY=wiFfYdH8Gipec8Eeeu0xXdbba9frFj0=OqFfea0dXdd9vqai=hGuQ8kuc9pgc9s8qqaq=dirpe0xb9q8qiLsFr0=vr0=vr0dc8meaabaqaciaacaGaaeqabaWaaeGaeaaakeaaimaacqWFZestaaa@3790@1	ℳ MathType@MTEF@5@5@+=feaafiart1ev1aaatCvAUfKttLearuWrP9MDH5MBPbIqV92AaeXatLxBI9gBamrtHrhAL1wy0L2yHvtyaeHbnfgDOvwBHrxAJfwnaebbnrfifHhDYfgasaacH8akY=wiFfYdH8Gipec8Eeeu0xXdbba9frFj0=OqFfea0dXdd9vqai=hGuQ8kuc9pgc9s8qqaq=dirpe0xb9q8qiLsFr0=vr0=vr0dc8meaabaqaciaacaGaaeqabaWaaeGaeaaakeaaimaacqWFZestaaa@3790@2	ℳ MathType@MTEF@5@5@+=feaafiart1ev1aaatCvAUfKttLearuWrP9MDH5MBPbIqV92AaeXatLxBI9gBamrtHrhAL1wy0L2yHvtyaeHbnfgDOvwBHrxAJfwnaebbnrfifHhDYfgasaacH8akY=wiFfYdH8Gipec8Eeeu0xXdbba9frFj0=OqFfea0dXdd9vqai=hGuQ8kuc9pgc9s8qqaq=dirpe0xb9q8qiLsFr0=vr0=vr0dc8meaabaqaciaacaGaaeqabaWaaeGaeaaakeaaimaacqWFZestaaa@3790@3
H	Int	hi−25,hi−33,hi−45,hi−73 MathType@MTEF@5@5@+=feaafiart1ev1aaatCvAUfKttLearuWrP9MDH5MBPbIqV92AaeXatLxBI9gBaebbnrfifHhDYfgasaacH8akY=wiFfYdH8Gipec8Eeeu0xXdbba9frFj0=OqFfea0dXdd9vqai=hGuQ8kuc9pgc9s8qqaq=dirpe0xb9q8qiLsFr0=vr0=vr0dc8meaabaqaciaacaGaaeqabaqabeGadaaakeaacqWGObaAdaqhaaWcbaGaemyAaKMaeyOeI0IaeGOmaidabaGaeGynaudaaOGaeiilaWIaemiAaG2aa0baaSqaaiabdMgaPjabgkHiTiabiodaZaqaaiabiodaZaaakiabcYcaSiabdIgaOnaaDaaaleaacqWGPbqAcqGHsislcqaI0aanaeaacqaI1aqnaaGccqGGSaalcqWGObaAdaqhaaWcbaGaemyAaKMaeyOeI0IaeG4naCdabaGaeG4mamdaaaaa@4652@	hi−23,hi−33,hi−43,hi+23,hi+43 MathType@MTEF@5@5@+=feaafiart1ev1aaatCvAUfKttLearuWrP9MDH5MBPbIqV92AaeXatLxBI9gBaebbnrfifHhDYfgasaacH8akY=wiFfYdH8Gipec8Eeeu0xXdbba9frFj0=OqFfea0dXdd9vqai=hGuQ8kuc9pgc9s8qqaq=dirpe0xb9q8qiLsFr0=vr0=vr0dc8meaabaqaciaacaGaaeqabaqabeGadaaakeaacqWGObaAdaqhaaWcbaGaemyAaKMaeyOeI0IaeGOmaidabaGaeG4mamdaaOGaeiilaWIaemiAaG2aa0baaSqaaiabdMgaPjabgkHiTiabiodaZaqaaiabiodaZaaakiabcYcaSiabdIgaOnaaDaaaleaacqWGPbqAcqGHsislcqaI0aanaeaacqaIZaWmaaGccqGGSaalcqWGObaAdaqhaaWcbaGaemyAaKMaey4kaSIaeGOmaidabaGaeG4mamdaaOGaeiilaWIaemiAaG2aa0baaSqaaiabdMgaPjabgUcaRiabisda0aqaaiabiodaZaaaaaa@4CCC@	hi+25,hi+35,hi+45 MathType@MTEF@5@5@+=feaafiart1ev1aaatCvAUfKttLearuWrP9MDH5MBPbIqV92AaeXatLxBI9gBaebbnrfifHhDYfgasaacH8akY=wiFfYdH8Gipec8Eeeu0xXdbba9frFj0=OqFfea0dXdd9vqai=hGuQ8kuc9pgc9s8qqaq=dirpe0xb9q8qiLsFr0=vr0=vr0dc8meaabaqaciaacaGaaeqabaqabeGadaaakeaacqWGObaAdaqhaaWcbaGaemyAaKMaey4kaSIaeGOmaidabaGaeGynaudaaOGaeiilaWIaemiAaG2aa0baaSqaaiabdMgaPjabgUcaRiabiodaZaqaaiabiwda1aaakiabcYcaSiabdIgaOnaaDaaaleaacqWGPbqAcqGHRaWkcqaI0aanaeaacqaI1aqnaaaaaa@3F8D@
	N1	hi−15,hi−25 MathType@MTEF@5@5@+=feaafiart1ev1aaatCvAUfKttLearuWrP9MDH5MBPbIqV92AaeXatLxBI9gBaebbnrfifHhDYfgasaacH8akY=wiFfYdH8Gipec8Eeeu0xXdbba9frFj0=OqFfea0dXdd9vqai=hGuQ8kuc9pgc9s8qqaq=dirpe0xb9q8qiLsFr0=vr0=vr0dc8meaabaqaciaacaGaaeqabaqabeGadaaakeaacqWGObaAdaqhaaWcbaGaemyAaKMaeyOeI0IaeGymaedabaGaeGynaudaaOGaeiilaWIaemiAaG2aa0baaSqaaiabdMgaPjabgkHiTiabikdaYaqaaiabiwda1aaaaaa@3904@	hi−15,hi+25 MathType@MTEF@5@5@+=feaafiart1ev1aaatCvAUfKttLearuWrP9MDH5MBPbIqV92AaeXatLxBI9gBaebbnrfifHhDYfgasaacH8akY=wiFfYdH8Gipec8Eeeu0xXdbba9frFj0=OqFfea0dXdd9vqai=hGuQ8kuc9pgc9s8qqaq=dirpe0xb9q8qiLsFr0=vr0=vr0dc8meaabaqaciaacaGaaeqabaqabeGadaaakeaacqWGObaAdaqhaaWcbaGaemyAaKMaeyOeI0IaeGymaedabaGaeGynaudaaOGaeiilaWIaemiAaG2aa0baaSqaaiabdMgaPjabgUcaRiabikdaYaqaaiabiwda1aaaaaa@38F9@	hi+25,hi+45 MathType@MTEF@5@5@+=feaafiart1ev1aaatCvAUfKttLearuWrP9MDH5MBPbIqV92AaeXatLxBI9gBaebbnrfifHhDYfgasaacH8akY=wiFfYdH8Gipec8Eeeu0xXdbba9frFj0=OqFfea0dXdd9vqai=hGuQ8kuc9pgc9s8qqaq=dirpe0xb9q8qiLsFr0=vr0=vr0dc8meaabaqaciaacaGaaeqabaqabeGadaaakeaacqWGObaAdaqhaaWcbaGaemyAaKMaey4kaSIaeGOmaidabaGaeGynaudaaOGaeiilaWIaemiAaG2aa0baaSqaaiabdMgaPjabgUcaRiabisda0aqaaiabiwda1aaaaaa@38F4@
	N2	hi−13,hi−23,hi−33 MathType@MTEF@5@5@+=feaafiart1ev1aaatCvAUfKttLearuWrP9MDH5MBPbIqV92AaeXatLxBI9gBaebbnrfifHhDYfgasaacH8akY=wiFfYdH8Gipec8Eeeu0xXdbba9frFj0=OqFfea0dXdd9vqai=hGuQ8kuc9pgc9s8qqaq=dirpe0xb9q8qiLsFr0=vr0=vr0dc8meaabaqaciaacaGaaeqabaqabeGadaaakeaacqWGObaAdaqhaaWcbaGaemyAaKMaeyOeI0IaeGymaedabaGaeG4mamdaaOGaeiilaWIaemiAaG2aa0baaSqaaiabdMgaPjabgkHiTiabikdaYaqaaiabiodaZaaakiabcYcaSiabdIgaOnaaDaaaleaacqWGPbqAcqGHsislcqaIZaWmaeaacqaIZaWmaaaaaa@3F9C@	hi−23,hi+23,hi+43 MathType@MTEF@5@5@+=feaafiart1ev1aaatCvAUfKttLearuWrP9MDH5MBPbIqV92AaeXatLxBI9gBaebbnrfifHhDYfgasaacH8akY=wiFfYdH8Gipec8Eeeu0xXdbba9frFj0=OqFfea0dXdd9vqai=hGuQ8kuc9pgc9s8qqaq=dirpe0xb9q8qiLsFr0=vr0=vr0dc8meaabaqaciaacaGaaeqabaqabeGadaaakeaacqWGObaAdaqhaaWcbaGaemyAaKMaeyOeI0IaeGOmaidabaGaeG4mamdaaOGaeiilaWIaemiAaG2aa0baaSqaaiabdMgaPjabgUcaRiabikdaYaqaaiabiodaZaaakiabcYcaSiabdIgaOnaaDaaaleaacqWGPbqAcqGHRaWkcqaI0aanaeaacqaIZaWmaaaaaa@3F8A@	hi+13,hi+23,hi+43 MathType@MTEF@5@5@+=feaafiart1ev1aaatCvAUfKttLearuWrP9MDH5MBPbIqV92AaeXatLxBI9gBaebbnrfifHhDYfgasaacH8akY=wiFfYdH8Gipec8Eeeu0xXdbba9frFj0=OqFfea0dXdd9vqai=hGuQ8kuc9pgc9s8qqaq=dirpe0xb9q8qiLsFr0=vr0=vr0dc8meaabaqaciaacaGaaeqabaqabeGadaaakeaacqWGObaAdaqhaaWcbaGaemyAaKMaey4kaSIaeGymaedabaGaeG4mamdaaOGaeiilaWIaemiAaG2aa0baaSqaaiabdMgaPjabgUcaRiabikdaYaqaaiabiodaZaaakiabcYcaSiabdIgaOnaaDaaaleaacqWGPbqAcqGHRaWkcqaI0aanaeaacqaIZaWmaaaaaa@3F7D@
	N3	hi−13,hi−23,hi−33 MathType@MTEF@5@5@+=feaafiart1ev1aaatCvAUfKttLearuWrP9MDH5MBPbIqV92AaeXatLxBI9gBaebbnrfifHhDYfgasaacH8akY=wiFfYdH8Gipec8Eeeu0xXdbba9frFj0=OqFfea0dXdd9vqai=hGuQ8kuc9pgc9s8qqaq=dirpe0xb9q8qiLsFr0=vr0=vr0dc8meaabaqaciaacaGaaeqabaqabeGadaaakeaacqWGObaAdaqhaaWcbaGaemyAaKMaeyOeI0IaeGymaedabaGaeG4mamdaaOGaeiilaWIaemiAaG2aa0baaSqaaiabdMgaPjabgkHiTiabikdaYaqaaiabiodaZaaakiabcYcaSiabdIgaOnaaDaaaleaacqWGPbqAcqGHsislcqaIZaWmaeaacqaIZaWmaaaaaa@3F9C@	hi−23,hi−33,hi+23 MathType@MTEF@5@5@+=feaafiart1ev1aaatCvAUfKttLearuWrP9MDH5MBPbIqV92AaeXatLxBI9gBaebbnrfifHhDYfgasaacH8akY=wiFfYdH8Gipec8Eeeu0xXdbba9frFj0=OqFfea0dXdd9vqai=hGuQ8kuc9pgc9s8qqaq=dirpe0xb9q8qiLsFr0=vr0=vr0dc8meaabaqaciaacaGaaeqabaqabeGadaaakeaacqWGObaAdaqhaaWcbaGaemyAaKMaeyOeI0IaeGOmaidabaGaeG4mamdaaOGaeiilaWIaemiAaG2aa0baaSqaaiabdMgaPjabgkHiTiabiodaZaqaaiabiodaZaaakiabcYcaSiabdIgaOnaaDaaaleaacqWGPbqAcqGHRaWkcqaIYaGmaeaacqaIZaWmaaaaaa@3F93@	hi+13,hi+23,hi+43 MathType@MTEF@5@5@+=feaafiart1ev1aaatCvAUfKttLearuWrP9MDH5MBPbIqV92AaeXatLxBI9gBaebbnrfifHhDYfgasaacH8akY=wiFfYdH8Gipec8Eeeu0xXdbba9frFj0=OqFfea0dXdd9vqai=hGuQ8kuc9pgc9s8qqaq=dirpe0xb9q8qiLsFr0=vr0=vr0dc8meaabaqaciaacaGaaeqabaqabeGadaaakeaacqWGObaAdaqhaaWcbaGaemyAaKMaey4kaSIaeGymaedabaGaeG4mamdaaOGaeiilaWIaemiAaG2aa0baaSqaaiabdMgaPjabgUcaRiabikdaYaqaaiabiodaZaaakiabcYcaSiabdIgaOnaaDaaaleaacqWGPbqAcqGHRaWkcqaI0aanaeaacqaIZaWmaaaaaa@3F7D@
	N4	hi−13,hi−23,hi−33 MathType@MTEF@5@5@+=feaafiart1ev1aaatCvAUfKttLearuWrP9MDH5MBPbIqV92AaeXatLxBI9gBaebbnrfifHhDYfgasaacH8akY=wiFfYdH8Gipec8Eeeu0xXdbba9frFj0=OqFfea0dXdd9vqai=hGuQ8kuc9pgc9s8qqaq=dirpe0xb9q8qiLsFr0=vr0=vr0dc8meaabaqaciaacaGaaeqabaqabeGadaaakeaacqWGObaAdaqhaaWcbaGaemyAaKMaeyOeI0IaeGymaedabaGaeG4mamdaaOGaeiilaWIaemiAaG2aa0baaSqaaiabdMgaPjabgkHiTiabikdaYaqaaiabiodaZaaakiabcYcaSiabdIgaOnaaDaaaleaacqWGPbqAcqGHsislcqaIZaWmaeaacqaIZaWmaaaaaa@3F9C@	hi−13,hi+23,hi+43 MathType@MTEF@5@5@+=feaafiart1ev1aaatCvAUfKttLearuWrP9MDH5MBPbIqV92AaeXatLxBI9gBaebbnrfifHhDYfgasaacH8akY=wiFfYdH8Gipec8Eeeu0xXdbba9frFj0=OqFfea0dXdd9vqai=hGuQ8kuc9pgc9s8qqaq=dirpe0xb9q8qiLsFr0=vr0=vr0dc8meaabaqaciaacaGaaeqabaqabeGadaaakeaacqWGObaAdaqhaaWcbaGaemyAaKMaeyOeI0IaeGymaedabaGaeG4mamdaaOGaeiilaWIaemiAaG2aa0baaSqaaiabdMgaPjabgUcaRiabikdaYaqaaiabiodaZaaakiabcYcaSiabdIgaOnaaDaaaleaacqWGPbqAcqGHRaWkcqaI0aanaeaacqaIZaWmaaaaaa@3F88@	hi+13,hi+23,hi+43 MathType@MTEF@5@5@+=feaafiart1ev1aaatCvAUfKttLearuWrP9MDH5MBPbIqV92AaeXatLxBI9gBaebbnrfifHhDYfgasaacH8akY=wiFfYdH8Gipec8Eeeu0xXdbba9frFj0=OqFfea0dXdd9vqai=hGuQ8kuc9pgc9s8qqaq=dirpe0xb9q8qiLsFr0=vr0=vr0dc8meaabaqaciaacaGaaeqabaqabeGadaaakeaacqWGObaAdaqhaaWcbaGaemyAaKMaey4kaSIaeGymaedabaGaeG4mamdaaOGaeiilaWIaemiAaG2aa0baaSqaaiabdMgaPjabgUcaRiabikdaYaqaaiabiodaZaaakiabcYcaSiabdIgaOnaaDaaaleaacqWGPbqAcqGHRaWkcqaI0aanaeaacqaIZaWmaaaaaa@3F7D@
	C1	hi−13,hi−23,hi−43 MathType@MTEF@5@5@+=feaafiart1ev1aaatCvAUfKttLearuWrP9MDH5MBPbIqV92AaeXatLxBI9gBaebbnrfifHhDYfgasaacH8akY=wiFfYdH8Gipec8Eeeu0xXdbba9frFj0=OqFfea0dXdd9vqai=hGuQ8kuc9pgc9s8qqaq=dirpe0xb9q8qiLsFr0=vr0=vr0dc8meaabaqaciaacaGaaeqabaqabeGadaaakeaacqWGObaAdaqhaaWcbaGaemyAaKMaeyOeI0IaeGymaedabaGaeG4mamdaaOGaeiilaWIaemiAaG2aa0baaSqaaiabdMgaPjabgkHiTiabikdaYaqaaiabiodaZaaakiabcYcaSiabdIgaOnaaDaaaleaacqWGPbqAcqGHsislcqaI0aanaeaacqaIZaWmaaaaaa@3F9E@	hi−23,hi−43,hi+13 MathType@MTEF@5@5@+=feaafiart1ev1aaatCvAUfKttLearuWrP9MDH5MBPbIqV92AaeXatLxBI9gBaebbnrfifHhDYfgasaacH8akY=wiFfYdH8Gipec8Eeeu0xXdbba9frFj0=OqFfea0dXdd9vqai=hGuQ8kuc9pgc9s8qqaq=dirpe0xb9q8qiLsFr0=vr0=vr0dc8meaabaqaciaacaGaaeqabaqabeGadaaakeaacqWGObaAdaqhaaWcbaGaemyAaKMaeyOeI0IaeGOmaidabaGaeG4mamdaaOGaeiilaWIaemiAaG2aa0baaSqaaiabdMgaPjabgkHiTiabisda0aqaaiabiodaZaaakiabcYcaSiabdIgaOnaaDaaaleaacqWGPbqAcqGHRaWkcqaIXaqmaeaacqaIZaWmaaaaaa@3F93@	hi+13,hi+23,hi+33 MathType@MTEF@5@5@+=feaafiart1ev1aaatCvAUfKttLearuWrP9MDH5MBPbIqV92AaeXatLxBI9gBaebbnrfifHhDYfgasaacH8akY=wiFfYdH8Gipec8Eeeu0xXdbba9frFj0=OqFfea0dXdd9vqai=hGuQ8kuc9pgc9s8qqaq=dirpe0xb9q8qiLsFr0=vr0=vr0dc8meaabaqaciaacaGaaeqabaqabeGadaaakeaacqWGObaAdaqhaaWcbaGaemyAaKMaey4kaSIaeGymaedabaGaeG4mamdaaOGaeiilaWIaemiAaG2aa0baaSqaaiabdMgaPjabgUcaRiabikdaYaqaaiabiodaZaaakiabcYcaSiabdIgaOnaaDaaaleaacqWGPbqAcqGHRaWkcqaIZaWmaeaacqaIZaWmaaaaaa@3F7B@
	C2	hi−23,hi−33,hi−43 MathType@MTEF@5@5@+=feaafiart1ev1aaatCvAUfKttLearuWrP9MDH5MBPbIqV92AaeXatLxBI9gBaebbnrfifHhDYfgasaacH8akY=wiFfYdH8Gipec8Eeeu0xXdbba9frFj0=OqFfea0dXdd9vqai=hGuQ8kuc9pgc9s8qqaq=dirpe0xb9q8qiLsFr0=vr0=vr0dc8meaabaqaciaacaGaaeqabaqabeGadaaakeaacqWGObaAdaqhaaWcbaGaemyAaKMaeyOeI0IaeGOmaidabaGaeG4mamdaaOGaeiilaWIaemiAaG2aa0baaSqaaiabdMgaPjabgkHiTiabiodaZaqaaiabiodaZaaakiabcYcaSiabdIgaOnaaDaaaleaacqWGPbqAcqGHsislcqaI0aanaeaacqaIZaWmaaaaaa@3FA2@	hi−23,hi−43,hi+13 MathType@MTEF@5@5@+=feaafiart1ev1aaatCvAUfKttLearuWrP9MDH5MBPbIqV92AaeXatLxBI9gBaebbnrfifHhDYfgasaacH8akY=wiFfYdH8Gipec8Eeeu0xXdbba9frFj0=OqFfea0dXdd9vqai=hGuQ8kuc9pgc9s8qqaq=dirpe0xb9q8qiLsFr0=vr0=vr0dc8meaabaqaciaacaGaaeqabaqabeGadaaakeaacqWGObaAdaqhaaWcbaGaemyAaKMaeyOeI0IaeGOmaidabaGaeG4mamdaaOGaeiilaWIaemiAaG2aa0baaSqaaiabdMgaPjabgkHiTiabisda0aqaaiabiodaZaaakiabcYcaSiabdIgaOnaaDaaaleaacqWGPbqAcqGHRaWkcqaIXaqmaeaacqaIZaWmaaaaaa@3F93@	hi+13,hi+23,hi+33 MathType@MTEF@5@5@+=feaafiart1ev1aaatCvAUfKttLearuWrP9MDH5MBPbIqV92AaeXatLxBI9gBaebbnrfifHhDYfgasaacH8akY=wiFfYdH8Gipec8Eeeu0xXdbba9frFj0=OqFfea0dXdd9vqai=hGuQ8kuc9pgc9s8qqaq=dirpe0xb9q8qiLsFr0=vr0=vr0dc8meaabaqaciaacaGaaeqabaqabeGadaaakeaacqWGObaAdaqhaaWcbaGaemyAaKMaey4kaSIaeGymaedabaGaeG4mamdaaOGaeiilaWIaemiAaG2aa0baaSqaaiabdMgaPjabgUcaRiabikdaYaqaaiabiodaZaaakiabcYcaSiabdIgaOnaaDaaaleaacqWGPbqAcqGHRaWkcqaIZaWmaeaacqaIZaWmaaaaaa@3F7B@

E	Int	hi−15,hi−25,hi−33 MathType@MTEF@5@5@+=feaafiart1ev1aaatCvAUfKttLearuWrP9MDH5MBPbIqV92AaeXatLxBI9gBaebbnrfifHhDYfgasaacH8akY=wiFfYdH8Gipec8Eeeu0xXdbba9frFj0=OqFfea0dXdd9vqai=hGuQ8kuc9pgc9s8qqaq=dirpe0xb9q8qiLsFr0=vr0=vr0dc8meaabaqaciaacaGaaeqabaqabeGadaaakeaacqWGObaAdaqhaaWcbaGaemyAaKMaeyOeI0IaeGymaedabaGaeGynaudaaOGaeiilaWIaemiAaG2aa0baaSqaaiabdMgaPjabgkHiTiabikdaYaqaaiabiwda1aaakiabcYcaSiabdIgaOnaaDaaaleaacqWGPbqAcqGHsislcqaIZaWmaeaacqaIZaWmaaaaaa@3FA4@	hi−13,hi−23,hi+13,hi+23 MathType@MTEF@5@5@+=feaafiart1ev1aaatCvAUfKttLearuWrP9MDH5MBPbIqV92AaeXatLxBI9gBaebbnrfifHhDYfgasaacH8akY=wiFfYdH8Gipec8Eeeu0xXdbba9frFj0=OqFfea0dXdd9vqai=hGuQ8kuc9pgc9s8qqaq=dirpe0xb9q8qiLsFr0=vr0=vr0dc8meaabaqaciaacaGaaeqabaqabeGadaaakeaacqWGObaAdaqhaaWcbaGaemyAaKMaeyOeI0IaeGymaedabaGaeG4mamdaaOGaeiilaWIaemiAaG2aa0baaSqaaiabdMgaPjabgkHiTiabikdaYaqaaiabiodaZaaakiabcYcaSiabdIgaOnaaDaaaleaacqWGPbqAcqGHRaWkcqaIXaqmaeaacqaIZaWmaaGccqGGSaalcqWGObaAdaqhaaWcbaGaemyAaKMaey4kaSIaeGOmaidabaGaeG4mamdaaaaa@4620@	hi+15,hi+25,hi+33 MathType@MTEF@5@5@+=feaafiart1ev1aaatCvAUfKttLearuWrP9MDH5MBPbIqV92AaeXatLxBI9gBaebbnrfifHhDYfgasaacH8akY=wiFfYdH8Gipec8Eeeu0xXdbba9frFj0=OqFfea0dXdd9vqai=hGuQ8kuc9pgc9s8qqaq=dirpe0xb9q8qiLsFr0=vr0=vr0dc8meaabaqaciaacaGaaeqabaqabeGadaaakeaacqWGObaAdaqhaaWcbaGaemyAaKMaey4kaSIaeGymaedabaGaeGynaudaaOGaeiilaWIaemiAaG2aa0baaSqaaiabdMgaPjabgUcaRiabikdaYaqaaiabiwda1aaakiabcYcaSiabdIgaOnaaDaaaleaacqWGPbqAcqGHRaWkcqaIZaWmaeaacqaIZaWmaaaaaa@3F83@
	N1	hi−15,hi−25 MathType@MTEF@5@5@+=feaafiart1ev1aaatCvAUfKttLearuWrP9MDH5MBPbIqV92AaeXatLxBI9gBaebbnrfifHhDYfgasaacH8akY=wiFfYdH8Gipec8Eeeu0xXdbba9frFj0=OqFfea0dXdd9vqai=hGuQ8kuc9pgc9s8qqaq=dirpe0xb9q8qiLsFr0=vr0=vr0dc8meaabaqaciaacaGaaeqabaqabeGadaaakeaacqWGObaAdaqhaaWcbaGaemyAaKMaeyOeI0IaeGymaedabaGaeGynaudaaOGaeiilaWIaemiAaG2aa0baaSqaaiabdMgaPjabgkHiTiabikdaYaqaaiabiwda1aaaaaa@3904@	hi−15,hi+15 MathType@MTEF@5@5@+=feaafiart1ev1aaatCvAUfKttLearuWrP9MDH5MBPbIqV92AaeXatLxBI9gBaebbnrfifHhDYfgasaacH8akY=wiFfYdH8Gipec8Eeeu0xXdbba9frFj0=OqFfea0dXdd9vqai=hGuQ8kuc9pgc9s8qqaq=dirpe0xb9q8qiLsFr0=vr0=vr0dc8meaabaqaciaacaGaaeqabaqabeGadaaakeaacqWGObaAdaqhaaWcbaGaemyAaKMaeyOeI0IaeGymaedabaGaeGynaudaaOGaeiilaWIaemiAaG2aa0baaSqaaiabdMgaPjabgUcaRiabigdaXaqaaiabiwda1aaaaaa@38F7@	hi+15,hi+25 MathType@MTEF@5@5@+=feaafiart1ev1aaatCvAUfKttLearuWrP9MDH5MBPbIqV92AaeXatLxBI9gBaebbnrfifHhDYfgasaacH8akY=wiFfYdH8Gipec8Eeeu0xXdbba9frFj0=OqFfea0dXdd9vqai=hGuQ8kuc9pgc9s8qqaq=dirpe0xb9q8qiLsFr0=vr0=vr0dc8meaabaqaciaacaGaaeqabaqabeGadaaakeaacqWGObaAdaqhaaWcbaGaemyAaKMaey4kaSIaeGymaedabaGaeGynaudaaOGaeiilaWIaemiAaG2aa0baaSqaaiabdMgaPjabgUcaRiabikdaYaqaaiabiwda1aaaaaa@38EE@
	N2	hi−15,hi−25 MathType@MTEF@5@5@+=feaafiart1ev1aaatCvAUfKttLearuWrP9MDH5MBPbIqV92AaeXatLxBI9gBaebbnrfifHhDYfgasaacH8akY=wiFfYdH8Gipec8Eeeu0xXdbba9frFj0=OqFfea0dXdd9vqai=hGuQ8kuc9pgc9s8qqaq=dirpe0xb9q8qiLsFr0=vr0=vr0dc8meaabaqaciaacaGaaeqabaqabeGadaaakeaacqWGObaAdaqhaaWcbaGaemyAaKMaeyOeI0IaeGymaedabaGaeGynaudaaOGaeiilaWIaemiAaG2aa0baaSqaaiabdMgaPjabgkHiTiabikdaYaqaaiabiwda1aaaaaa@3904@	hi−15,hi+25 MathType@MTEF@5@5@+=feaafiart1ev1aaatCvAUfKttLearuWrP9MDH5MBPbIqV92AaeXatLxBI9gBaebbnrfifHhDYfgasaacH8akY=wiFfYdH8Gipec8Eeeu0xXdbba9frFj0=OqFfea0dXdd9vqai=hGuQ8kuc9pgc9s8qqaq=dirpe0xb9q8qiLsFr0=vr0=vr0dc8meaabaqaciaacaGaaeqabaqabeGadaaakeaacqWGObaAdaqhaaWcbaGaemyAaKMaeyOeI0IaeGymaedabaGaeGynaudaaOGaeiilaWIaemiAaG2aa0baaSqaaiabdMgaPjabgUcaRiabikdaYaqaaiabiwda1aaaaaa@38F9@	hi+15,hi+25 MathType@MTEF@5@5@+=feaafiart1ev1aaatCvAUfKttLearuWrP9MDH5MBPbIqV92AaeXatLxBI9gBaebbnrfifHhDYfgasaacH8akY=wiFfYdH8Gipec8Eeeu0xXdbba9frFj0=OqFfea0dXdd9vqai=hGuQ8kuc9pgc9s8qqaq=dirpe0xb9q8qiLsFr0=vr0=vr0dc8meaabaqaciaacaGaaeqabaqabeGadaaakeaacqWGObaAdaqhaaWcbaGaemyAaKMaey4kaSIaeGymaedabaGaeGynaudaaOGaeiilaWIaemiAaG2aa0baaSqaaiabdMgaPjabgUcaRiabikdaYaqaaiabiwda1aaaaaa@38EE@
	C1	hi−13,hi−33,hi−43 MathType@MTEF@5@5@+=feaafiart1ev1aaatCvAUfKttLearuWrP9MDH5MBPbIqV92AaeXatLxBI9gBaebbnrfifHhDYfgasaacH8akY=wiFfYdH8Gipec8Eeeu0xXdbba9frFj0=OqFfea0dXdd9vqai=hGuQ8kuc9pgc9s8qqaq=dirpe0xb9q8qiLsFr0=vr0=vr0dc8meaabaqaciaacaGaaeqabaqabeGadaaakeaacqWGObaAdaqhaaWcbaGaemyAaKMaeyOeI0IaeGymaedabaGaeG4mamdaaOGaeiilaWIaemiAaG2aa0baaSqaaiabdMgaPjabgkHiTiabiodaZaqaaiabiodaZaaakiabcYcaSiabdIgaOnaaDaaaleaacqWGPbqAcqGHsislcqaI0aanaeaacqaIZaWmaaaaaa@3FA0@	hi−13,hi−33,hi+13 MathType@MTEF@5@5@+=feaafiart1ev1aaatCvAUfKttLearuWrP9MDH5MBPbIqV92AaeXatLxBI9gBaebbnrfifHhDYfgasaacH8akY=wiFfYdH8Gipec8Eeeu0xXdbba9frFj0=OqFfea0dXdd9vqai=hGuQ8kuc9pgc9s8qqaq=dirpe0xb9q8qiLsFr0=vr0=vr0dc8meaabaqaciaacaGaaeqabaqabeGadaaakeaacqWGObaAdaqhaaWcbaGaemyAaKMaeyOeI0IaeGymaedabaGaeG4mamdaaOGaeiilaWIaemiAaG2aa0baaSqaaiabdMgaPjabgkHiTiabiodaZaqaaiabiodaZaaakiabcYcaSiabdIgaOnaaDaaaleaacqWGPbqAcqGHRaWkcqaIXaqmaeaacqaIZaWmaaaaaa@3F8F@	hi+13,hi+23,hi+33 MathType@MTEF@5@5@+=feaafiart1ev1aaatCvAUfKttLearuWrP9MDH5MBPbIqV92AaeXatLxBI9gBaebbnrfifHhDYfgasaacH8akY=wiFfYdH8Gipec8Eeeu0xXdbba9frFj0=OqFfea0dXdd9vqai=hGuQ8kuc9pgc9s8qqaq=dirpe0xb9q8qiLsFr0=vr0=vr0dc8meaabaqaciaacaGaaeqabaqabeGadaaakeaacqWGObaAdaqhaaWcbaGaemyAaKMaey4kaSIaeGymaedabaGaeG4mamdaaOGaeiilaWIaemiAaG2aa0baaSqaaiabdMgaPjabgUcaRiabikdaYaqaaiabiodaZaaakiabcYcaSiabdIgaOnaaDaaaleaacqWGPbqAcqGHRaWkcqaIZaWmaeaacqaIZaWmaaaaaa@3F7B@
	C2	hi−15,hi−25 MathType@MTEF@5@5@+=feaafiart1ev1aaatCvAUfKttLearuWrP9MDH5MBPbIqV92AaeXatLxBI9gBaebbnrfifHhDYfgasaacH8akY=wiFfYdH8Gipec8Eeeu0xXdbba9frFj0=OqFfea0dXdd9vqai=hGuQ8kuc9pgc9s8qqaq=dirpe0xb9q8qiLsFr0=vr0=vr0dc8meaabaqaciaacaGaaeqabaqabeGadaaakeaacqWGObaAdaqhaaWcbaGaemyAaKMaeyOeI0IaeGymaedabaGaeGynaudaaOGaeiilaWIaemiAaG2aa0baaSqaaiabdMgaPjabgkHiTiabikdaYaqaaiabiwda1aaaaaa@3904@	hi−15,hi+15 MathType@MTEF@5@5@+=feaafiart1ev1aaatCvAUfKttLearuWrP9MDH5MBPbIqV92AaeXatLxBI9gBaebbnrfifHhDYfgasaacH8akY=wiFfYdH8Gipec8Eeeu0xXdbba9frFj0=OqFfea0dXdd9vqai=hGuQ8kuc9pgc9s8qqaq=dirpe0xb9q8qiLsFr0=vr0=vr0dc8meaabaqaciaacaGaaeqabaqabeGadaaakeaacqWGObaAdaqhaaWcbaGaemyAaKMaeyOeI0IaeGymaedabaGaeGynaudaaOGaeiilaWIaemiAaG2aa0baaSqaaiabdMgaPjabgUcaRiabigdaXaqaaiabiwda1aaaaaa@38F7@	hi+15,hi+25 MathType@MTEF@5@5@+=feaafiart1ev1aaatCvAUfKttLearuWrP9MDH5MBPbIqV92AaeXatLxBI9gBaebbnrfifHhDYfgasaacH8akY=wiFfYdH8Gipec8Eeeu0xXdbba9frFj0=OqFfea0dXdd9vqai=hGuQ8kuc9pgc9s8qqaq=dirpe0xb9q8qiLsFr0=vr0=vr0dc8meaabaqaciaacaGaaeqabaqabeGadaaakeaacqWGObaAdaqhaaWcbaGaemyAaKMaey4kaSIaeGymaedabaGaeGynaudaaOGaeiilaWIaemiAaG2aa0baaSqaaiabdMgaPjabgUcaRiabikdaYaqaaiabiwda1aaaaaa@38EE@

L	Int	hi−15,hi−25,hi−33,hi−43 MathType@MTEF@5@5@+=feaafiart1ev1aaatCvAUfKttLearuWrP9MDH5MBPbIqV92AaeXatLxBI9gBaebbnrfifHhDYfgasaacH8akY=wiFfYdH8Gipec8Eeeu0xXdbba9frFj0=OqFfea0dXdd9vqai=hGuQ8kuc9pgc9s8qqaq=dirpe0xb9q8qiLsFr0=vr0=vr0dc8meaabaqaciaacaGaaeqabaqabeGadaaakeaacqWGObaAdaqhaaWcbaGaemyAaKMaeyOeI0IaeGymaedabaGaeGynaudaaOGaeiilaWIaemiAaG2aa0baaSqaaiabdMgaPjabgkHiTiabikdaYaqaaiabiwda1aaakiabcYcaSiabdIgaOnaaDaaaleaacqWGPbqAcqGHsislcqaIZaWmaeaacqaIZaWmaaGccqGGSaalcqWGObaAdaqhaaWcbaGaemyAaKMaeyOeI0IaeGinaqdabaGaeG4mamdaaaaa@4646@	hi−15,hi−23,hi+15,hi+23 MathType@MTEF@5@5@+=feaafiart1ev1aaatCvAUfKttLearuWrP9MDH5MBPbIqV92AaeXatLxBI9gBaebbnrfifHhDYfgasaacH8akY=wiFfYdH8Gipec8Eeeu0xXdbba9frFj0=OqFfea0dXdd9vqai=hGuQ8kuc9pgc9s8qqaq=dirpe0xb9q8qiLsFr0=vr0=vr0dc8meaabaqaciaacaGaaeqabaqabeGadaaakeaacqWGObaAdaqhaaWcbaGaemyAaKMaeyOeI0IaeGymaedabaGaeGynaudaaOGaeiilaWIaemiAaG2aa0baaSqaaiabdMgaPjabgkHiTiabikdaYaqaaiabiodaZaaakiabcYcaSiabdIgaOnaaDaaaleaacqWGPbqAcqGHRaWkcqaIXaqmaeaacqaI1aqnaaGccqGGSaalcqWGObaAdaqhaaWcbaGaemyAaKMaey4kaSIaeGOmaidabaGaeG4mamdaaaaa@4628@	hi+15,hi+25,hi+33,hi+43 MathType@MTEF@5@5@+=feaafiart1ev1aaatCvAUfKttLearuWrP9MDH5MBPbIqV92AaeXatLxBI9gBaebbnrfifHhDYfgasaacH8akY=wiFfYdH8Gipec8Eeeu0xXdbba9frFj0=OqFfea0dXdd9vqai=hGuQ8kuc9pgc9s8qqaq=dirpe0xb9q8qiLsFr0=vr0=vr0dc8meaabaqaciaacaGaaeqabaqabeGadaaakeaacqWGObaAdaqhaaWcbaGaemyAaKMaey4kaSIaeGymaedabaGaeGynaudaaOGaeiilaWIaemiAaG2aa0baaSqaaiabdMgaPjabgUcaRiabikdaYaqaaiabiwda1aaakiabcYcaSiabdIgaOnaaDaaaleaacqWGPbqAcqGHRaWkcqaIZaWmaeaacqaIZaWmaaGccqGGSaalcqWGObaAdaqhaaWcbaGaemyAaKMaey4kaSIaeGinaqdabaGaeG4mamdaaaaa@461A@
	N1	hi−15,hi−25,hi−33 MathType@MTEF@5@5@+=feaafiart1ev1aaatCvAUfKttLearuWrP9MDH5MBPbIqV92AaeXatLxBI9gBaebbnrfifHhDYfgasaacH8akY=wiFfYdH8Gipec8Eeeu0xXdbba9frFj0=OqFfea0dXdd9vqai=hGuQ8kuc9pgc9s8qqaq=dirpe0xb9q8qiLsFr0=vr0=vr0dc8meaabaqaciaacaGaaeqabaqabeGadaaakeaacqWGObaAdaqhaaWcbaGaemyAaKMaeyOeI0IaeGymaedabaGaeGynaudaaOGaeiilaWIaemiAaG2aa0baaSqaaiabdMgaPjabgkHiTiabikdaYaqaaiabiwda1aaakiabcYcaSiabdIgaOnaaDaaaleaacqWGPbqAcqGHsislcqaIZaWmaeaacqaIZaWmaaaaaa@3FA4@	hi−15,hi−25,hi+13 MathType@MTEF@5@5@+=feaafiart1ev1aaatCvAUfKttLearuWrP9MDH5MBPbIqV92AaeXatLxBI9gBaebbnrfifHhDYfgasaacH8akY=wiFfYdH8Gipec8Eeeu0xXdbba9frFj0=OqFfea0dXdd9vqai=hGuQ8kuc9pgc9s8qqaq=dirpe0xb9q8qiLsFr0=vr0=vr0dc8meaabaqaciaacaGaaeqabaqabeGadaaakeaacqWGObaAdaqhaaWcbaGaemyAaKMaeyOeI0IaeGymaedabaGaeGynaudaaOGaeiilaWIaemiAaG2aa0baaSqaaiabdMgaPjabgkHiTiabikdaYaqaaiabiwda1aaakiabcYcaSiabdIgaOnaaDaaaleaacqWGPbqAcqGHRaWkcqaIXaqmaeaacqaIZaWmaaaaaa@3F95@	hi+15,hi+25,hi+33 MathType@MTEF@5@5@+=feaafiart1ev1aaatCvAUfKttLearuWrP9MDH5MBPbIqV92AaeXatLxBI9gBaebbnrfifHhDYfgasaacH8akY=wiFfYdH8Gipec8Eeeu0xXdbba9frFj0=OqFfea0dXdd9vqai=hGuQ8kuc9pgc9s8qqaq=dirpe0xb9q8qiLsFr0=vr0=vr0dc8meaabaqaciaacaGaaeqabaqabeGadaaakeaacqWGObaAdaqhaaWcbaGaemyAaKMaey4kaSIaeGymaedabaGaeGynaudaaOGaeiilaWIaemiAaG2aa0baaSqaaiabdMgaPjabgUcaRiabikdaYaqaaiabiwda1aaakiabcYcaSiabdIgaOnaaDaaaleaacqWGPbqAcqGHRaWkcqaIZaWmaeaacqaIZaWmaaaaaa@3F83@
	N2	hi−15,hi−23,hi−43 MathType@MTEF@5@5@+=feaafiart1ev1aaatCvAUfKttLearuWrP9MDH5MBPbIqV92AaeXatLxBI9gBaebbnrfifHhDYfgasaacH8akY=wiFfYdH8Gipec8Eeeu0xXdbba9frFj0=OqFfea0dXdd9vqai=hGuQ8kuc9pgc9s8qqaq=dirpe0xb9q8qiLsFr0=vr0=vr0dc8meaabaqaciaacaGaaeqabaqabeGadaaakeaacqWGObaAdaqhaaWcbaGaemyAaKMaeyOeI0IaeGymaedabaGaeGynaudaaOGaeiilaWIaemiAaG2aa0baaSqaaiabdMgaPjabgkHiTiabikdaYaqaaiabiodaZaaakiabcYcaSiabdIgaOnaaDaaaleaacqWGPbqAcqGHsislcqaI0aanaeaacqaIZaWmaaaaaa@3FA2@	hi−15,hi−23,hi+13 MathType@MTEF@5@5@+=feaafiart1ev1aaatCvAUfKttLearuWrP9MDH5MBPbIqV92AaeXatLxBI9gBaebbnrfifHhDYfgasaacH8akY=wiFfYdH8Gipec8Eeeu0xXdbba9frFj0=OqFfea0dXdd9vqai=hGuQ8kuc9pgc9s8qqaq=dirpe0xb9q8qiLsFr0=vr0=vr0dc8meaabaqaciaacaGaaeqabaqabeGadaaakeaacqWGObaAdaqhaaWcbaGaemyAaKMaeyOeI0IaeGymaedabaGaeGynaudaaOGaeiilaWIaemiAaG2aa0baaSqaaiabdMgaPjabgkHiTiabikdaYaqaaiabiodaZaaakiabcYcaSiabdIgaOnaaDaaaleaacqWGPbqAcqGHRaWkcqaIXaqmaeaacqaIZaWmaaaaaa@3F91@	hi+15,hi+23,hi+43 MathType@MTEF@5@5@+=feaafiart1ev1aaatCvAUfKttLearuWrP9MDH5MBPbIqV92AaeXatLxBI9gBaebbnrfifHhDYfgasaacH8akY=wiFfYdH8Gipec8Eeeu0xXdbba9frFj0=OqFfea0dXdd9vqai=hGuQ8kuc9pgc9s8qqaq=dirpe0xb9q8qiLsFr0=vr0=vr0dc8meaabaqaciaacaGaaeqabaqabeGadaaakeaacqWGObaAdaqhaaWcbaGaemyAaKMaey4kaSIaeGymaedabaGaeGynaudaaOGaeiilaWIaemiAaG2aa0baaSqaaiabdMgaPjabgUcaRiabikdaYaqaaiabiodaZaaakiabcYcaSiabdIgaOnaaDaaaleaacqWGPbqAcqGHRaWkcqaI0aanaeaacqaIZaWmaaaaaa@3F81@
	N3	hi−15,hi−23,hi−33 MathType@MTEF@5@5@+=feaafiart1ev1aaatCvAUfKttLearuWrP9MDH5MBPbIqV92AaeXatLxBI9gBaebbnrfifHhDYfgasaacH8akY=wiFfYdH8Gipec8Eeeu0xXdbba9frFj0=OqFfea0dXdd9vqai=hGuQ8kuc9pgc9s8qqaq=dirpe0xb9q8qiLsFr0=vr0=vr0dc8meaabaqaciaacaGaaeqabaqabeGadaaakeaacqWGObaAdaqhaaWcbaGaemyAaKMaeyOeI0IaeGymaedabaGaeGynaudaaOGaeiilaWIaemiAaG2aa0baaSqaaiabdMgaPjabgkHiTiabikdaYaqaaiabiodaZaaakiabcYcaSiabdIgaOnaaDaaaleaacqWGPbqAcqGHsislcqaIZaWmaeaacqaIZaWmaaaaaa@3FA0@	hi−15,hi−23,hi+13 MathType@MTEF@5@5@+=feaafiart1ev1aaatCvAUfKttLearuWrP9MDH5MBPbIqV92AaeXatLxBI9gBaebbnrfifHhDYfgasaacH8akY=wiFfYdH8Gipec8Eeeu0xXdbba9frFj0=OqFfea0dXdd9vqai=hGuQ8kuc9pgc9s8qqaq=dirpe0xb9q8qiLsFr0=vr0=vr0dc8meaabaqaciaacaGaaeqabaqabeGadaaakeaacqWGObaAdaqhaaWcbaGaemyAaKMaeyOeI0IaeGymaedabaGaeGynaudaaOGaeiilaWIaemiAaG2aa0baaSqaaiabdMgaPjabgkHiTiabikdaYaqaaiabiodaZaaakiabcYcaSiabdIgaOnaaDaaaleaacqWGPbqAcqGHRaWkcqaIXaqmaeaacqaIZaWmaaaaaa@3F91@	hi+15,hi+23,hi+33 MathType@MTEF@5@5@+=feaafiart1ev1aaatCvAUfKttLearuWrP9MDH5MBPbIqV92AaeXatLxBI9gBaebbnrfifHhDYfgasaacH8akY=wiFfYdH8Gipec8Eeeu0xXdbba9frFj0=OqFfea0dXdd9vqai=hGuQ8kuc9pgc9s8qqaq=dirpe0xb9q8qiLsFr0=vr0=vr0dc8meaabaqaciaacaGaaeqabaqabeGadaaakeaacqWGObaAdaqhaaWcbaGaemyAaKMaey4kaSIaeGymaedabaGaeGynaudaaOGaeiilaWIaemiAaG2aa0baaSqaaiabdMgaPjabgUcaRiabikdaYaqaaiabiodaZaaakiabcYcaSiabdIgaOnaaDaaaleaacqWGPbqAcqGHRaWkcqaIZaWmaeaacqaIZaWmaaaaaa@3F7F@
	N4	hi−15,hi−23,hi−33 MathType@MTEF@5@5@+=feaafiart1ev1aaatCvAUfKttLearuWrP9MDH5MBPbIqV92AaeXatLxBI9gBaebbnrfifHhDYfgasaacH8akY=wiFfYdH8Gipec8Eeeu0xXdbba9frFj0=OqFfea0dXdd9vqai=hGuQ8kuc9pgc9s8qqaq=dirpe0xb9q8qiLsFr0=vr0=vr0dc8meaabaqaciaacaGaaeqabaqabeGadaaakeaacqWGObaAdaqhaaWcbaGaemyAaKMaeyOeI0IaeGymaedabaGaeGynaudaaOGaeiilaWIaemiAaG2aa0baaSqaaiabdMgaPjabgkHiTiabikdaYaqaaiabiodaZaaakiabcYcaSiabdIgaOnaaDaaaleaacqWGPbqAcqGHsislcqaIZaWmaeaacqaIZaWmaaaaaa@3FA0@	hi−15,hi−23,hi+13 MathType@MTEF@5@5@+=feaafiart1ev1aaatCvAUfKttLearuWrP9MDH5MBPbIqV92AaeXatLxBI9gBaebbnrfifHhDYfgasaacH8akY=wiFfYdH8Gipec8Eeeu0xXdbba9frFj0=OqFfea0dXdd9vqai=hGuQ8kuc9pgc9s8qqaq=dirpe0xb9q8qiLsFr0=vr0=vr0dc8meaabaqaciaacaGaaeqabaqabeGadaaakeaacqWGObaAdaqhaaWcbaGaemyAaKMaeyOeI0IaeGymaedabaGaeGynaudaaOGaeiilaWIaemiAaG2aa0baaSqaaiabdMgaPjabgkHiTiabikdaYaqaaiabiodaZaaakiabcYcaSiabdIgaOnaaDaaaleaacqWGPbqAcqGHRaWkcqaIXaqmaeaacqaIZaWmaaaaaa@3F91@	hi+15,hi+23,hi+33 MathType@MTEF@5@5@+=feaafiart1ev1aaatCvAUfKttLearuWrP9MDH5MBPbIqV92AaeXatLxBI9gBaebbnrfifHhDYfgasaacH8akY=wiFfYdH8Gipec8Eeeu0xXdbba9frFj0=OqFfea0dXdd9vqai=hGuQ8kuc9pgc9s8qqaq=dirpe0xb9q8qiLsFr0=vr0=vr0dc8meaabaqaciaacaGaaeqabaqabeGadaaakeaacqWGObaAdaqhaaWcbaGaemyAaKMaey4kaSIaeGymaedabaGaeGynaudaaOGaeiilaWIaemiAaG2aa0baaSqaaiabdMgaPjabgUcaRiabikdaYaqaaiabiodaZaaakiabcYcaSiabdIgaOnaaDaaaleaacqWGPbqAcqGHRaWkcqaIZaWmaeaacqaIZaWmaaaaaa@3F7F@
	C1	hi−15,hi−25,hi−33 MathType@MTEF@5@5@+=feaafiart1ev1aaatCvAUfKttLearuWrP9MDH5MBPbIqV92AaeXatLxBI9gBaebbnrfifHhDYfgasaacH8akY=wiFfYdH8Gipec8Eeeu0xXdbba9frFj0=OqFfea0dXdd9vqai=hGuQ8kuc9pgc9s8qqaq=dirpe0xb9q8qiLsFr0=vr0=vr0dc8meaabaqaciaacaGaaeqabaqabeGadaaakeaacqWGObaAdaqhaaWcbaGaemyAaKMaeyOeI0IaeGymaedabaGaeGynaudaaOGaeiilaWIaemiAaG2aa0baaSqaaiabdMgaPjabgkHiTiabikdaYaqaaiabiwda1aaakiabcYcaSiabdIgaOnaaDaaaleaacqWGPbqAcqGHsislcqaIZaWmaeaacqaIZaWmaaaaaa@3FA4@	hi−15,hi−25,hi+13 MathType@MTEF@5@5@+=feaafiart1ev1aaatCvAUfKttLearuWrP9MDH5MBPbIqV92AaeXatLxBI9gBaebbnrfifHhDYfgasaacH8akY=wiFfYdH8Gipec8Eeeu0xXdbba9frFj0=OqFfea0dXdd9vqai=hGuQ8kuc9pgc9s8qqaq=dirpe0xb9q8qiLsFr0=vr0=vr0dc8meaabaqaciaacaGaaeqabaqabeGadaaakeaacqWGObaAdaqhaaWcbaGaemyAaKMaeyOeI0IaeGymaedabaGaeGynaudaaOGaeiilaWIaemiAaG2aa0baaSqaaiabdMgaPjabgkHiTiabikdaYaqaaiabiwda1aaakiabcYcaSiabdIgaOnaaDaaaleaacqWGPbqAcqGHRaWkcqaIXaqmaeaacqaIZaWmaaaaaa@3F95@	hi+15,hi+25,hi+33 MathType@MTEF@5@5@+=feaafiart1ev1aaatCvAUfKttLearuWrP9MDH5MBPbIqV92AaeXatLxBI9gBaebbnrfifHhDYfgasaacH8akY=wiFfYdH8Gipec8Eeeu0xXdbba9frFj0=OqFfea0dXdd9vqai=hGuQ8kuc9pgc9s8qqaq=dirpe0xb9q8qiLsFr0=vr0=vr0dc8meaabaqaciaacaGaaeqabaqabeGadaaakeaacqWGObaAdaqhaaWcbaGaemyAaKMaey4kaSIaeGymaedabaGaeGynaudaaOGaeiilaWIaemiAaG2aa0baaSqaaiabdMgaPjabgUcaRiabikdaYaqaaiabiwda1aaakiabcYcaSiabdIgaOnaaDaaaleaacqWGPbqAcqGHRaWkcqaIZaWmaeaacqaIZaWmaaaaaa@3F83@
	C2	hi−15,hi−25 MathType@MTEF@5@5@+=feaafiart1ev1aaatCvAUfKttLearuWrP9MDH5MBPbIqV92AaeXatLxBI9gBaebbnrfifHhDYfgasaacH8akY=wiFfYdH8Gipec8Eeeu0xXdbba9frFj0=OqFfea0dXdd9vqai=hGuQ8kuc9pgc9s8qqaq=dirpe0xb9q8qiLsFr0=vr0=vr0dc8meaabaqaciaacaGaaeqabaqabeGadaaakeaacqWGObaAdaqhaaWcbaGaemyAaKMaeyOeI0IaeGymaedabaGaeGynaudaaOGaeiilaWIaemiAaG2aa0baaSqaaiabdMgaPjabgkHiTiabikdaYaqaaiabiwda1aaaaaa@3904@	hi−15,hi+35 MathType@MTEF@5@5@+=feaafiart1ev1aaatCvAUfKttLearuWrP9MDH5MBPbIqV92AaeXatLxBI9gBaebbnrfifHhDYfgasaacH8akY=wiFfYdH8Gipec8Eeeu0xXdbba9frFj0=OqFfea0dXdd9vqai=hGuQ8kuc9pgc9s8qqaq=dirpe0xb9q8qiLsFr0=vr0=vr0dc8meaabaqaciaacaGaaeqabaqabeGadaaakeaacqWGObaAdaqhaaWcbaGaemyAaKMaeyOeI0IaeGymaedabaGaeGynaudaaOGaeiilaWIaemiAaG2aa0baaSqaaiabdMgaPjabgUcaRiabiodaZaqaaiabiwda1aaaaaa@38FB@	hi+15,hi+25 MathType@MTEF@5@5@+=feaafiart1ev1aaatCvAUfKttLearuWrP9MDH5MBPbIqV92AaeXatLxBI9gBaebbnrfifHhDYfgasaacH8akY=wiFfYdH8Gipec8Eeeu0xXdbba9frFj0=OqFfea0dXdd9vqai=hGuQ8kuc9pgc9s8qqaq=dirpe0xb9q8qiLsFr0=vr0=vr0dc8meaabaqaciaacaGaaeqabaqabeGadaaakeaacqWGObaAdaqhaaWcbaGaemyAaKMaey4kaSIaeGymaedabaGaeGynaudaaOGaeiilaWIaemiAaG2aa0baaSqaaiabdMgaPjabgUcaRiabikdaYaqaaiabiwda1aaaaaa@38EE@
	C3	hi−13,hi−23,hi−33 MathType@MTEF@5@5@+=feaafiart1ev1aaatCvAUfKttLearuWrP9MDH5MBPbIqV92AaeXatLxBI9gBaebbnrfifHhDYfgasaacH8akY=wiFfYdH8Gipec8Eeeu0xXdbba9frFj0=OqFfea0dXdd9vqai=hGuQ8kuc9pgc9s8qqaq=dirpe0xb9q8qiLsFr0=vr0=vr0dc8meaabaqaciaacaGaaeqabaqabeGadaaakeaacqWGObaAdaqhaaWcbaGaemyAaKMaeyOeI0IaeGymaedabaGaeG4mamdaaOGaeiilaWIaemiAaG2aa0baaSqaaiabdMgaPjabgkHiTiabikdaYaqaaiabiodaZaaakiabcYcaSiabdIgaOnaaDaaaleaacqWGPbqAcqGHsislcqaIZaWmaeaacqaIZaWmaaaaaa@3F9C@	hi−13,hi+13,hi+23 MathType@MTEF@5@5@+=feaafiart1ev1aaatCvAUfKttLearuWrP9MDH5MBPbIqV92AaeXatLxBI9gBaebbnrfifHhDYfgasaacH8akY=wiFfYdH8Gipec8Eeeu0xXdbba9frFj0=OqFfea0dXdd9vqai=hGuQ8kuc9pgc9s8qqaq=dirpe0xb9q8qiLsFr0=vr0=vr0dc8meaabaqaciaacaGaaeqabaqabeGadaaakeaacqWGObaAdaqhaaWcbaGaemyAaKMaeyOeI0IaeGymaedabaGaeG4mamdaaOGaeiilaWIaemiAaG2aa0baaSqaaiabdMgaPjabgUcaRiabigdaXaqaaiabiodaZaaakiabcYcaSiabdIgaOnaaDaaaleaacqWGPbqAcqGHRaWkcqaIYaGmaeaacqaIZaWmaaaaaa@3F82@	hi+13,hi+23,hi+33 MathType@MTEF@5@5@+=feaafiart1ev1aaatCvAUfKttLearuWrP9MDH5MBPbIqV92AaeXatLxBI9gBaebbnrfifHhDYfgasaacH8akY=wiFfYdH8Gipec8Eeeu0xXdbba9frFj0=OqFfea0dXdd9vqai=hGuQ8kuc9pgc9s8qqaq=dirpe0xb9q8qiLsFr0=vr0=vr0dc8meaabaqaciaacaGaaeqabaqabeGadaaakeaacqWGObaAdaqhaaWcbaGaemyAaKMaey4kaSIaeGymaedabaGaeG4mamdaaOGaeiilaWIaemiAaG2aa0baaSqaaiabdMgaPjabgUcaRiabikdaYaqaaiabiodaZaaakiabcYcaSiabdIgaOnaaDaaaleaacqWGPbqAcqGHRaWkcqaIZaWmaeaacqaIZaWmaaaaaa@3F7B@
	C4	hi−13,hi−23,hi−33 MathType@MTEF@5@5@+=feaafiart1ev1aaatCvAUfKttLearuWrP9MDH5MBPbIqV92AaeXatLxBI9gBaebbnrfifHhDYfgasaacH8akY=wiFfYdH8Gipec8Eeeu0xXdbba9frFj0=OqFfea0dXdd9vqai=hGuQ8kuc9pgc9s8qqaq=dirpe0xb9q8qiLsFr0=vr0=vr0dc8meaabaqaciaacaGaaeqabaqabeGadaaakeaacqWGObaAdaqhaaWcbaGaemyAaKMaeyOeI0IaeGymaedabaGaeG4mamdaaOGaeiilaWIaemiAaG2aa0baaSqaaiabdMgaPjabgkHiTiabikdaYaqaaiabiodaZaaakiabcYcaSiabdIgaOnaaDaaaleaacqWGPbqAcqGHsislcqaIZaWmaeaacqaIZaWmaaaaaa@3F9C@	hi−23,hi−33,hi+13 MathType@MTEF@5@5@+=feaafiart1ev1aaatCvAUfKttLearuWrP9MDH5MBPbIqV92AaeXatLxBI9gBaebbnrfifHhDYfgasaacH8akY=wiFfYdH8Gipec8Eeeu0xXdbba9frFj0=OqFfea0dXdd9vqai=hGuQ8kuc9pgc9s8qqaq=dirpe0xb9q8qiLsFr0=vr0=vr0dc8meaabaqaciaacaGaaeqabaqabeGadaaakeaacqWGObaAdaqhaaWcbaGaemyAaKMaeyOeI0IaeGOmaidabaGaeG4mamdaaOGaeiilaWIaemiAaG2aa0baaSqaaiabdMgaPjabgkHiTiabiodaZaqaaiabiodaZaaakiabcYcaSiabdIgaOnaaDaaaleaacqWGPbqAcqGHRaWkcqaIXaqmaeaacqaIZaWmaaaaaa@3F91@	hi+13,hi+23,hi+33 MathType@MTEF@5@5@+=feaafiart1ev1aaatCvAUfKttLearuWrP9MDH5MBPbIqV92AaeXatLxBI9gBaebbnrfifHhDYfgasaacH8akY=wiFfYdH8Gipec8Eeeu0xXdbba9frFj0=OqFfea0dXdd9vqai=hGuQ8kuc9pgc9s8qqaq=dirpe0xb9q8qiLsFr0=vr0=vr0dc8meaabaqaciaacaGaaeqabaqabeGadaaakeaacqWGObaAdaqhaaWcbaGaemyAaKMaey4kaSIaeGymaedabaGaeG4mamdaaOGaeiilaWIaemiAaG2aa0baaSqaaiabdMgaPjabgUcaRiabikdaYaqaaiabiodaZaaakiabcYcaSiabdIgaOnaaDaaaleaacqWGPbqAcqGHRaWkcqaIZaWmaeaacqaIZaWmaaaaaa@3F7B@

**Table 8:** Prediction sensitivity measures, *Q*.(%) evaluated on the EVA set under the single-sequence condition.

Sensitivity	*Q*_3_(%)	*Q*_*α*_(%)	*Q*_*β*_(%)	*Q*_*L*_(%)
BSPSS	68.400	63.203	36.737	**82.167**
IPSSP	**70.300**	**65.934**	**45.445**	81.280

**Table 9:** Segment border sensitivity values, *Q*._-*sb*_(%), evaluated on the EVA set under the single-sequence condition.

Sensitivity	*Q*_3_*sb*_(%)	*Q*_*α*_*sb*_(%)	*Q*_*β*_*sb*_(%)	*Q*_*L*_*sb*_(%)
BSPSS	62.207	52.634	24.215	**81.903**
IPSSP	**63.883**	**55.669**	**32.754**	80.303

**Table 10:** Prediction specificity measures, *SP*.(%), evaluated on the EVA set under the single-sequence condition.

Specificity	*SP*._*α*_(%)	*SP*._*β*_(%)	*SP*_*L*_(%)
BSPSS	68.636	**59.728**	69.832
IPSSP	**72.132**	59.203	**72.002**

**Table 11:** Matthew's correlation coefficient values, *C*., evaluated on the EVA set under the single-sequence condition.

MCC	*C*_*α*_	*C*_*β*_	*C*_*L*_
BSPSS	0.5195	0.3849	0.4468
IPSSP	**0.5638**	**0.4312**	**0.4764**

**Table 12:** Prediction sensitivity measures, *Q*.(%), analyzed with respect to three conversion rules and length adjustments, evaluated on the EVA set under the single-sequence condition.

Sensitivity	Q_3_(%)	*Q*_*α*_(%)	*Q*_*β*_(%)	*Q*_*L*_(%)
BSPSS Rule 1	65.177	65.655	38.844	76.644
BSPSS Rule 2	65.175	65.640	38.814	76.658
BSPSS Rule 3	67.218	64.048	38.071	80.491
BSPSS Rule 1 + Length adj	68.060	63.775	37.022	81.378
BSPSS Rule 2 + Length adj	68.078	63.793	37.017	81.399
BSPSS Rule 3 + Length adj	68.400	63.203	36.737	**82.167**
IPSSP Rule 1	67.415	**68.115**	46.386	76.340
IPSSP Rule 2	67.421	68.089	**46.395**	76.363
IPSSP Rule 3	69.096	66.559	45.319	79.893
IPSSP Rule 1 + Length adj	70.027	66.557	45.588	80.577
IPSSP Rule 2 + Length adj	70.036	66.554	45.559	80.602
IPSSP Rule 3 + Length adj	**70.300**	65.934	45.445	81.280

**Table 13:** Performances of the BSPSS, IPSSP with dependency models, ℳ
 MathType@MTEF@5@5@+=feaafiart1ev1aaatCvAUfKttLearuWrP9MDH5MBPbIqV92AaeXatLxBI9gBamrtHrhAL1wy0L2yHvtyaeHbnfgDOvwBHrxAJfwnaebbnrfifHhDYfgasaacH8akY=wiFfYdH8Gipec8Eeeu0xXdbba9frFj0=OqFfea0dXdd9vqai=hGuQ8kuc9pgc9s8qqaq=dirpe0xb9q8qiLsFr0=vr0=vr0dc8meaabaqaciaacaGaaeqabaWaaeGaeaaakeaaimaacqWFZestaaa@3790@_1_, ℳ
 MathType@MTEF@5@5@+=feaafiart1ev1aaatCvAUfKttLearuWrP9MDH5MBPbIqV92AaeXatLxBI9gBamrtHrhAL1wy0L2yHvtyaeHbnfgDOvwBHrxAJfwnaebbnrfifHhDYfgasaacH8akY=wiFfYdH8Gipec8Eeeu0xXdbba9frFj0=OqFfea0dXdd9vqai=hGuQ8kuc9pgc9s8qqaq=dirpe0xb9q8qiLsFr0=vr0=vr0dc8meaabaqaciaacaGaaeqabaWaaeGaeaaakeaaimaacqWFZestaaa@3790@_2_, ℳ
 MathType@MTEF@5@5@+=feaafiart1ev1aaatCvAUfKttLearuWrP9MDH5MBPbIqV92AaeXatLxBI9gBamrtHrhAL1wy0L2yHvtyaeHbnfgDOvwBHrxAJfwnaebbnrfifHhDYfgasaacH8akY=wiFfYdH8Gipec8Eeeu0xXdbba9frFj0=OqFfea0dXdd9vqai=hGuQ8kuc9pgc9s8qqaq=dirpe0xb9q8qiLsFr0=vr0=vr0dc8meaabaqaciaacaGaaeqabaWaaeGaeaaakeaaimaacqWFZestaaa@3790@_3_, and IPSSP with the combined model, ℳ
 MathType@MTEF@5@5@+=feaafiart1ev1aaatCvAUfKttLearuWrP9MDH5MBPbIqV92AaeXatLxBI9gBamrtHrhAL1wy0L2yHvtyaeHbnfgDOvwBHrxAJfwnaebbnrfifHhDYfgasaacH8akY=wiFfYdH8Gipec8Eeeu0xXdbba9frFj0=OqFfea0dXdd9vqai=hGuQ8kuc9pgc9s8qqaq=dirpe0xb9q8qiLsFr0=vr0=vr0dc8meaabaqaciaacaGaaeqabaWaaeGaeaaakeaaimaacqWFZestaaa@3790@_*C *_(obtained using an averaging filter), evaluated on the EVA set under the single-sequence condition.

Sensitivity	*Q*_3_(%)	*Q*_*α*_(%)	*Q*_*β*_(%)	*Q*_*L*_(%)
BSPSS	65.175	65.640	38.814	**76.658**
IPSSP-ℳ MathType@MTEF@5@5@+=feaafiart1ev1aaatCvAUfKttLearuWrP9MDH5MBPbIqV92AaeXatLxBI9gBamrtHrhAL1wy0L2yHvtyaeHbnfgDOvwBHrxAJfwnaebbnrfifHhDYfgasaacH8akY=wiFfYdH8Gipec8Eeeu0xXdbba9frFj0=OqFfea0dXdd9vqai=hGuQ8kuc9pgc9s8qqaq=dirpe0xb9q8qiLsFr0=vr0=vr0dc8meaabaqaciaacaGaaeqabaWaaeGaeaaakeaaimaacqWFZestaaa@3790@_1_	65.968	66.199	45.387	75.043
IPSSP-ℳ MathType@MTEF@5@5@+=feaafiart1ev1aaatCvAUfKttLearuWrP9MDH5MBPbIqV92AaeXatLxBI9gBamrtHrhAL1wy0L2yHvtyaeHbnfgDOvwBHrxAJfwnaebbnrfifHhDYfgasaacH8akY=wiFfYdH8Gipec8Eeeu0xXdbba9frFj0=OqFfea0dXdd9vqai=hGuQ8kuc9pgc9s8qqaq=dirpe0xb9q8qiLsFr0=vr0=vr0dc8meaabaqaciaacaGaaeqabaWaaeGaeaaakeaaimaacqWFZestaaa@3790@_2_	66.003	66.606	**46.952**	74.108
IPSSP-ℳ MathType@MTEF@5@5@+=feaafiart1ev1aaatCvAUfKttLearuWrP9MDH5MBPbIqV92AaeXatLxBI9gBamrtHrhAL1wy0L2yHvtyaeHbnfgDOvwBHrxAJfwnaebbnrfifHhDYfgasaacH8akY=wiFfYdH8Gipec8Eeeu0xXdbba9frFj0=OqFfea0dXdd9vqai=hGuQ8kuc9pgc9s8qqaq=dirpe0xb9q8qiLsFr0=vr0=vr0dc8meaabaqaciaacaGaaeqabaWaaeGaeaaakeaaimaacqWFZestaaa@3790@_3_	66.315	67.012	45.005	75.364
IPSSP-ℳ MathType@MTEF@5@5@+=feaafiart1ev1aaatCvAUfKttLearuWrP9MDH5MBPbIqV92AaeXatLxBI9gBamrtHrhAL1wy0L2yHvtyaeHbnfgDOvwBHrxAJfwnaebbnrfifHhDYfgasaacH8akY=wiFfYdH8Gipec8Eeeu0xXdbba9frFj0=OqFfea0dXdd9vqai=hGuQ8kuc9pgc9s8qqaq=dirpe0xb9q8qiLsFr0=vr0=vr0dc8meaabaqaciaacaGaaeqabaWaaeGaeaaakeaaimaacqWFZestaaa@3790@_*C*_	**67.421**	**68.089**	46.395	76.363

**Table 14:** Comparison of prediction sensitivity measures, *Q*.(%), for BSPSS, IPSSP and PSSP (the method that does not use iterative training), evaluated on the EVA set under the single-sequence condition.

Sensitivity	Q_3_(%)	*Q*_*α*_(%)	*Q*_*β*_(%)	*Q*_*L*_(%)
BSPSS	65.175	65.640	38.814	76.658
IPSSP	**67.421**	**68.089**	**46.395**	76.363
PSSP	66.840	66.945	44.566	**76.761**

**Table 15:** Prediction sensitivity measures, *Q*.(%), evaluated on the CASP6 targets.

Sensitivity	*Q*_3_(%)	*Q*_*α*_(%)	*Q*_*β*_(%)	*Q*_*L*_(%)
BSPSS	66.541	75.177	41.743	72.696
IPSSP	**67.899**	74.984	46.087	**73.755**
PSIPRED	67.680	**76.066**	**52.032**	69.028

**Table 16:** Matthew's correlation coefficient values, evaluated on the CASP6 targets.

MCC	*C*_*α*_(%)	*C*_*β*_(%)	*C*_*L*_(%)
BSPSS	0.5403	0.4354	0.4457
IPSSP	**0.5657**	0.4486	**0.4696**
PSIPRED	0.5465	**0.4801**	0.4646
