# Supplementary material for: Protein secondary structure prediction for a single-sequence using hidden semi-Markov models
Source: BMC Bioinformatics. 2006 Mar 30;7:178. doi: 10.1186/1471-2105-7-178 (PMC1479840; doi:10.1186/1471-2105-7-178)
Supplement: Additional File 1 — Segment Overlap Score. In this file, the performances of the methods BSPSS and IPSSP are evaluated and compared on the Segment Overlap (SOV) measure, which is based on the average overlap between the observed and the predicted segments. [file 1471-2105-7-178-S1.pdf]

Segment OVerlap Score

The Segment OVerlap score (SOV) is based on the average overlap between the observed and the predicted segments instead of the average per-residue accuracy [1,2]. The SOV measures provide more elaborate scoring, in which the predictions that have high per-residue accuracy but deviate from experimental segment length distributions are assigned lower scores. For instance, the definition of the SOV measure for α-helices is as follows:

$$SOV_\alpha = \frac{1}{N_\alpha} \sum_{S_\alpha} \frac{minOV(s_1, s_2) + \delta(s_1, s_2)}{maxOV(s_1, s_2)}. \tag{1}$$

Here, s_1 and s_2 are the observed and predicted secondary structure segments in the α-helix state; S_α is the number of all segment pairs (s_1, s_2), where s_1 and s_2 have at least one residue in α-helix state in common, $minOV(s_1, s_2)$ is the length of the actual overlap of s_1 and s_2 and $maxOV(s_1, s_2)$ is the length of the total extent for which either of the segments s_1 or s_2 has a residue in α-helix state. N_α is the total number of amino acid residues observed in the α-helix conformation. The definition of $\delta(s1, s2)$ is as follows [2]:

$$\delta(s_1, s_2) = min \left\{ \begin{array}{c} maxOV(s_1, s_2) - minOV(s_1, s_2) \\ minOV(s_1, s_2) \\ int(0.5 \times len(s_1)) \\ int(0.5 \times len(s_2)). \end{array} \right\} \tag{2}$$

Here, $len(s_1)$ is the number of amino acid residues in the segment s_1. The segment overlap measure for all three states, $SOV_3(\%)$, is similar to the $Q_3(\%)$ sensitivity measure:

$$SOV_3(\%) = \frac{1}{N} \left(\sum_{i \in H,E,L} \sum_{S(i)} \left[\frac{minOV(s_1, s_2) + \delta(s_1, s_2)}{maxOV(s_1, s_2)} \times len(s_1) \right] \right) \times 100 \tag{3}$$

Here, s_1 and s_2 are the observed and predicted secondary structure segments in state i. N is the total length of proteins under consideration. The SOV scores of BSPSS and IPSSP are evaluated and compared on the set of "sequence-unique" proteins derived from the PDB database. The set can be downloaded from the EVA server [3]. In terms of the Segment Overlap scores, IPSSP performs uniformly better than BSPSS under the single-sequence condition (Table 1).

Table 1: Segment overlap measures, $SOV_.(\%)$, for BSPSS and IPSSP evaluated on the EVA set under the single-sequence condition. To reduce 8 states to 3, the third conversion rule (CK mapping: H to H, E to E and all other states to L) is used with the length adjustments.

SOV	$SOV_3(\%)$	$SOV_\alpha(\%)$	$SOV_\beta(\%)$	$SOV_L(\%)$
BSPSS	61.064	67.418	45.940	62.514
IPSSP	**63.662**	**69.765**	**54.682**	**63.171**

References

1. Rost B, Sander C, Schneider R: **Redefining the goals of protein secondary structure prediction**. *J. Mol. Biol.* 1994, **235**:13–26.

2. Zemla A, Venclovas C, Fidelis K, Rost B: **A modified definition of SOV, a segment-based measure for protein secondary structure prediction assessment**. *Proteins* 1999, **34**:220–223.

3. **EVA Set**. [http://cubic.bioc.columbia.edu/eva/doc/ftp.html].
