# Supplementary material for: Protein secondary structure prediction for a single-sequence using hidden semi-Markov models
Source: BMC Bioinformatics. 2006 Mar 30;7:178. doi: 10.1186/1471-2105-7-178 (PMC1479840; doi:10.1186/1471-2105-7-178)
Supplement: Additional File 2 — Reliability Measures. In this file, the performances of the methods BSPSS and IPSSP are evaluated and compared on two reliability measures: the prediction confidence and the percentage of predicted positions. Both measures are computed with respect to the prediction threshold. [file 1471-2105-7-178-S2.pdf]

Reliability Measures

To estimate the confidence in predictions, we computed the overall sensitivity (Q_3) as a function of the probability assigned to the predicted state at each position. For instance, at a threshold prediction probability of 0.6, with 57% of sequence positions in this category, we achieved a Q_3 of 78.8%. On the other hand, at a threshold prediction probability of 0.8, with 19% of positions in this category, we obtained a Q_3 of 89.9% (Table 1). In terms of the prediction confidence and the positions covered, IPSSP performs uniformly better than BSPSS.

Table 1: Percentage of true positives for predictions made in a set of positions having the *a posteriori* probability of the predicted state above the threshold. To reduce 8 states to 3, the second conversion rule (H, G to H, E, B to E and all other states to L) is used.

Prediction Confidence (% Positions)			
Prediction Threshold	0.4	0.6	0.8
BSPSS	0.6639 (95.505)	0.7857 (48.580)	0.8977 (12.888)
IPSSP	**0.6829 (97.108)**	**0.7884 (57.022)**	**0.8995 (19.168)**
